# Eco-evolutionary significance of “loners”

**DOI:** 10.1371/journal.pbio.3000642

**Published:** 2020-03-19

**Authors:** Fernando W. Rossine, Ricardo Martinez-Garcia, Allyson E. Sgro, Thomas Gregor, Corina E. Tarnita

**Affiliations:** 1 Department of Ecology and Evolutionary Biology, Princeton University, Princeton, New Jersey, United States of America; 2 ICTP-South American Institute for Fundamental Research and Instituto de Fisica Teorica da UNESP, Sao Paulo, SP Brazil; 3 Joseph Henry Laboratories of Physics and Lewis-Sigler Institute for Integrative Genomics, Princeton University, Princeton, New Jersey, United States of America; 4 Department of Biomedical Engineering and the Biological Design Center, Boston University, Boston, Massachusetts, United States of America; 5 Department of Developmental and Stem Cell Biology, UMR3738, Institut Pasteur, Paris, France; University of Michigan, UNITED STATES

## Abstract

Loners—individuals out of sync with a coordinated majority—occur frequently in nature. Are loners incidental byproducts of large-scale coordination attempts, or are they part of a mosaic of life-history strategies? Here, we provide empirical evidence of naturally occurring heritable variation in loner behavior in the model social amoeba *Dictyostelium discoideum*. We propose that *Dictyostelium* loners—cells that do not join the multicellular life stage—arise from a dynamic population-partitioning process, the result of each cell making a stochastic, signal-based decision. We find evidence that this imperfectly synchronized multicellular development is affected by both abiotic (environmental porosity) and biotic (signaling) factors. Finally, we predict theoretically that when a pair of strains differing in their partitioning behavior coaggregate, cross-signaling impacts slime-mold diversity across spatiotemporal scales. Our findings suggest that loners could be critical to understanding collective and social behaviors, multicellular development, and ecological dynamics in *D*. *discoideum*. More broadly, across taxa, imperfect coordination of collective behaviors might be adaptive by enabling diversification of life-history strategies.

## Abstract 2

Loners (individuals out of sync with a coordinated majority) occur frequently in nature and are generally assumed to be incidental by-products of imperfect coordination attempts. Experimental and theoretical work on the slime mold Dictyostelium discoideum suggests that "lonerism" might actually be an alternative life-history strategy.

## Introduction

Collective behaviors, in which a large number of individuals exhibit some degree of behavioral coordination, are frequent across the tree of life and across spatiotemporal scales: from microbial aggregates to the great wildebeest migration, from locust swarming to synchronized bamboo flowering, from fish schooling to mechanical adaptation in honeybee clusters [1–9]. Intriguingly, however, such coordination is sometimes imperfect, and “out-of-sync” individuals (henceforth loners) have been reported in several of these systems. For instance, in locusts, population crowding prompts a transition from a solitary phase, in which individuals repel each other, to a gregarious phase, in which they attract each other. Experiments show, however, that not all individuals undergo this transition, even if exposed to long periods of crowding [4]. In wildebeest, hundreds of thousands of individuals coordinate with each other and organize herding migrations, but resident populations that fail to migrate also exist [2]. Similarly, wildebeest calving times are highly coordinated, but some fraction of the calves are born outside the calving period [2]. In bamboo, individuals predominantly flower in synchronized masts, but sporadic out-of-sync events have also been recorded [3].

The roots of such imperfect coordination, and hence the mechanisms underlying the emergence of loners, will undoubtedly differ across systems (for example, genetic factors, ad hoc strategic choices, stochasticity in the response to external cues). Nevertheless, the occurrence of imperfect coordination across such different systems and scales raises fundamental questions about its causes and consequences. Are loners mistakes—merely inevitable byproducts of large-scale coordination attempts—or can they, at least in some systems, be a variable trait that selection can shape with potential ecological consequences? Theoretical investigations of such loner behaviors have been sparse, but the handful of existing studies have suggested that, at least in some systems, they could be a means of spatiotemporal niche partitioning [10] that promotes diversity [11,12]. However, despite this theoretically established potential, the variability and heritability of loner behaviors have not been characterized in natural populations. Thus, there exists no empirical evidence, in any system, that loners are anything more than chance stragglers, lacking an avenue for selection to act on them.

The cellular slime mold *Dictyostelium discoideum* is an ideal system in which to experimentally characterize loner behaviors. Its life cycle comprises a unicellular feeding stage and a starvation-induced multicellular stage—the result of a developmental process involving coordinated cell aggregation, which culminates in the production of starvation-resistant spores [13]. There has been extensive progress in understanding this multicellular stage [1,13,14], but less attention has been paid to the potential role of asocial aspects, such as the nonaggregating solitary loner cells, in development. Loners die under sustained starvation, but they persist temporarily [15]; if food is replenished, they eat and divide, and their progeny subsequently recapitulate the multicellular development [11]. In addition, it has been shown that strains with key genes knocked out can have different loner behaviors [10]. Altogether, these observations suggest that loners could indeed be part of a life-history strategy in *D*. *discoideum*. However, to fully establish this, one needs to show (a) that natural populations of *D*. *discoideum* have heritable variation in loner behavior (i.e., heritability) and (b) that the loner behavior of different strains affects strain relative abundances in their natural environments (i.e., that in natural environments, there are fitness differences between strains with different loner behaviors). Here, we first establish (a) by experimentally inspecting loner behaviors in naturally co-occurring strains. Subsequently, by combining experiments and modeling, we suggest candidate self-organization rules underlying the aggregator–loner partitioning and explore their interaction with the abiotic (substrate porosity) and biotic (other co-occurring strains) environments. Finally, by modeling the interactions between coaggregating strains, we investigate potential long-term (over many growth–starvation cycles) ecological consequences of the aggregator–loner partitioning, which also provides some theoretical insights into (b).

## Results and discussion

### The aggregator–loner partitioning is heritable and context-dependent

**Fig 1:**
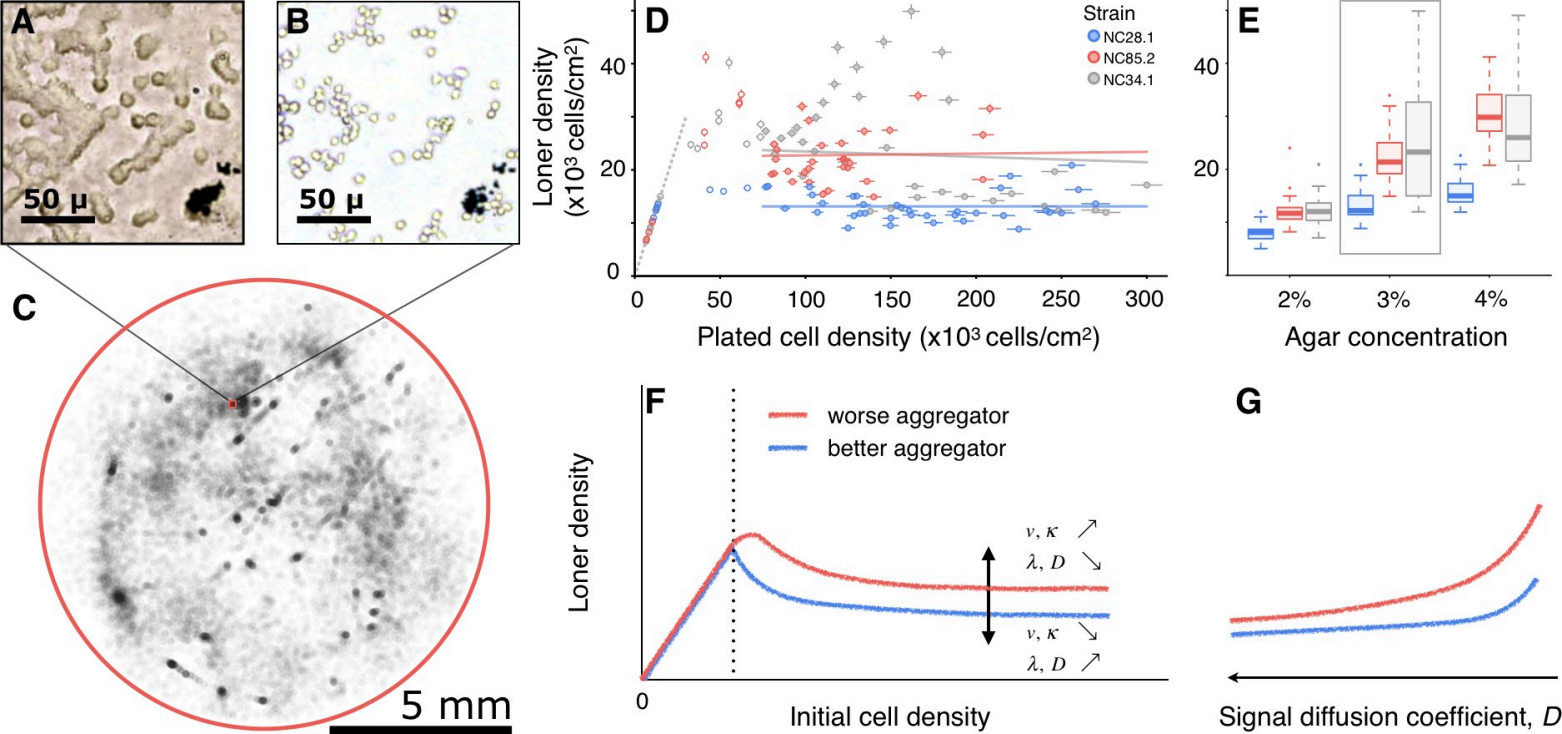
Loners are a heritable component of *D*. *discoideum* fitness. (A, B) After aggregation, loner cells are hard to individualize (A), but boundaries between cells become visible after processing (B). (C) Map of the position of each loner cell in an experiment (NC85.2 developing in 3% agar). Red square marks the region shown in A and B. (D) Loner densities of 3 strains as a function of initial plated density in 3% agar. Error bars, independent estimates of counting error (see Materials and Methods). Lines correspond to linear regressions using only high-initial–density data points (solid circles, >75,000 cells/cm^2^). (E) Loner densities from experiments with high initial cell density as a function of substrate agar concentration (y-axis same as in D). Boxes, interquartile ranges; horizontal lines, medians; whiskers, 1.5× interquartile range from the median; points, outliers. Strain NC28.1 always left fewer loners (*t* test, *p* < 0.001). Values inside the frame correspond to data used in (D). (F, G) Schematic of model results showing loner densities as a function of initial cell density (F) and signal diffusion coefficient (G). The y-axis in (G) is the same as in (F).

To determine whether the loner behavior is heritable—and, thus, whether there is the potential for natural selection to act on it—we developed an experimental protocol to identify and quantify numbers of individual loner cells (Fig 1A and 1B), which permitted us to characterize their spatial distribution (Fig 1C and S1 Fig), quantify their density (Fig 1D, 1E and S2 Fig), and set tight bounds on our measurement errors (S3A and S3B Fig). Importantly, we worked with 3 natural isolates [16,17] (i.e., strains or genetic variants) that were collected from the same location to ensure that observed behaviors of individual strains were not an artifact of lab rearing and that observed behavioral differences among strains reflected naturally co-occurring strategies. When homogeneously plated cells of a given strain were left to starve and undergo multicellular development, loner cells were found throughout aggregation territories, with a higher density at territory borders than in the immediate surroundings of aggregation centers (Fig 1C, S1 Fig, S2 Fig and S1–S6 Videos). In repeated experiments under controlled conditions, loner densities of a given strain fell consistently within a conserved distribution (framed portion of Fig 1E, independently replicated in Figs 3 and 4B); moreover, the loner distributions of some strains were significantly different in their mean and variance (compare strains NC28.1 and NC85.2 in Fig 1D and framed portion of Fig 1E). These findings demonstrate that the aggregator–loner partitioning behavior is heritable and that, therefore, it has the potential to be shaped by selection.

To characterize the self-organization process underlying the partitioning, we must first determine whether a cell’s decision to commit to aggregation or remain a loner is context-independent [10,11] or whether it depends on external factors. Here, we use “decision-making” in the broad sense employed in animal behavior literature: a cell makes a decision when it performs an action from a set of possible actions, potentially based on a sensorial input [18–20]. If such decision-making is context-independent (no external cues), then loner density should increase linearly with the density of initially plated cells, i.e., the heritable quantity would be the fraction of loners, as previously posited [10,11]. Instead, we found a nonlinear dependence: at low initial densities, cells were too sparse for aggregation to occur, and all cells remained loners [1,21]; above a threshold, aggregation occurred with increasing efficiency, and loner densities decreased; surprisingly, at high initial cell densities, loner densities plateaued (Fig 1D and S3C–S3K Fig). Thus, past a range of initial densities, it is the number (or density) and not the fraction of loners that is heritable, suggesting an underlying cell decision-making process that is fundamentally different from a context-independent stochastic switch. Henceforth, we define the aggregation performance of a strain to be the value of this plateau: we say that one strain is a better aggregator than another if the former plateaus at a lower value than the latter. When we varied the porosity of the agar substrate—a proxy for an important environmental characteristic for this soil-dwelling amoeba—less porous (more concentrated) agar yielded higher loner densities (Fig 1E) and differentially affected *D*. *discoideum* strains by enhancing the difference between strains that leave fewer loners (“better aggregators”) and those that leave more loners (“worse aggregators”). Interestingly, the agar porosity affects not only the mean number of loners but also the variance (Fig 1E). Altogether, these findings demonstrate that the heritable aggregator–loner partitioning is also context-dependent—the result of a density-dependent decision-making process that interacts with the abiotic environment.

### An abiotically modulated quorum-based stochastic process can reproduce the observed aggregator–loner partitioning

To investigate the properties of this decision-making process, we constructed a spatially explicit individual-based model (S4 Fig; see Materials and Methods) around one central hypothesis: that stochasticity in the responses of individual cells to surrounding population densities (i.e., to quorum sensing) leads to imperfect synchronization in the timing of the developmental program and gives rise to the observed loners.

Consistent with our experimental design, we started with a population of cells immediately after food exhaustion and assumed them to be in a preaggregating state, *P*. Following experimental results that show a reduced mobility of vegetative cells in crowded populations [22], we assume that *P*-cells do not move. *P*-cells emit extracellular signaling molecules at a strain-specific rate *γ*. The signal diffuses with diffusion coefficient *D* and serves a quorum-sensing purpose [1,23] that regulates the stochastic transition to the aggregating state, *A*: when the signal perceived by a cell exceeds the strain-specific sensitivity threshold *θ* (i.e., the quorum is met), our model assumes that cell to have a strain-specific probability per unit time *λ* of becoming an aggregating *A*-cell. *A*-cells continue to emit signal and move in straight displacements towards the aggregation center with constant, strain-specific velocity *ν*. At the center, cells become multicellular (*M*-state), and they stop moving and emitting signal.

Because our model focuses on the population partitioning, it deliberately simplifies certain dynamics that may underlie the *P*-to-*A* transition and the aggregation dynamics. For instance, before aggregating, cells must sequentially starve, become excitable by cyclic adenosine monophosphate (cAMP) [1,24,25], and finally chemotax [1]. Some of these steps, such as cell starvation or the activation of conditioned medium factor (CMF) production during early starvation, are irreversible. Others, such as establishing the cAMP relay or chemotaxing, are reversible. For simplicity, we bind together all these processes into a single, irreversible transition between the preaggregating and aggregating states (*P*-to-*A* transition). Therefore, in our model, the nondeterministic factors that are associated with all these processes and that could influence the timing of the overarching *P*-to-*A* transition are subsumed into *λ*. Thus, the stochasticity imparted by *λ* models both inherently stochastic factors affecting process waiting times (such as receptor binding or transcription bursts) and variability in the physiological state of the cells (such as nutritional state or chromatin configuration). As a result of the stochasticity, the times at which *P*-cells transition to the *A*-state follow a distribution, the width of which sets the degree of synchronization in the aggregation process. Higher values of *λ* lead to narrower distributions and thus more synchronized *P*-to-*A* transitions, whereas lower values of *λ* lead to wider distributions and a more asynchronous process. Importantly, we do not impose restrictions on the nature of the signaling molecule or on the sensing mechanism, and the model employs this molecule broadly to fulfill both a traditional [23,26] and a dynamical quorum-sensing role [1].

Our model makes further simplifications to the *P*-to-*A* transition and the aggregation dynamics. For example, acquiring aggregation competence is likely related to continuous changes in the levels of expression of certain key proteins. Similarly, acquiring aggregation competence is likely related to continuous changes in the levels of expression of certain key proteins. Because we are primarily interested in the time at which cells start responding to aggregation, this continuous sensitization with potentially many steps can, for simplicity, be characterized by its mean completion time (which corresponds to the time at which the *P*-to-*A* transition occurs in our model and is thus related to *λ*). Another simplifying assumption regards the dynamics of cAMP. During aggregation, cells establish traveling waves of cAMP on which they chemotax towards endogenously emerging aggregation centers [1,27–29]. In our model, the location of the aggregation center is a fixed external parameter and not an emergent feature of the aggregation process [30]; moreover, we do not account for the spatiotemporal complexities of *D*. *discoideum* movement during aggregation, such as the tortuosity in single-cell trajectories caused by imperfect chemotaxis [31] or the cAMP-induced aggregation patterns [32–34]. However, the net effect of all these features is to regulate the duration of cell migration towards the aggregation center, which can be represented in our model by changing cell velocity. Finally, for computational convenience, our model simplifies various additional sources of stochasticity that arise during the developmental process, such as the existence of multiple aggregation territories and the variable boundary shapes between them. Some sources of stochasticity, such as the short-range and long-range dynamics that shape the formation of territory boundaries, are expected to qualitatively affect the model results such as the variability in loner densities. But other sources, such as multiple aggregation territories, are not expected to qualitatively change the model results because the use of periodic boundary conditions in our single-territory model (see Materials and Methods) mimics the existence of signal fluxes through territory boundaries in a multiterritory setup.

**Fig 2:**
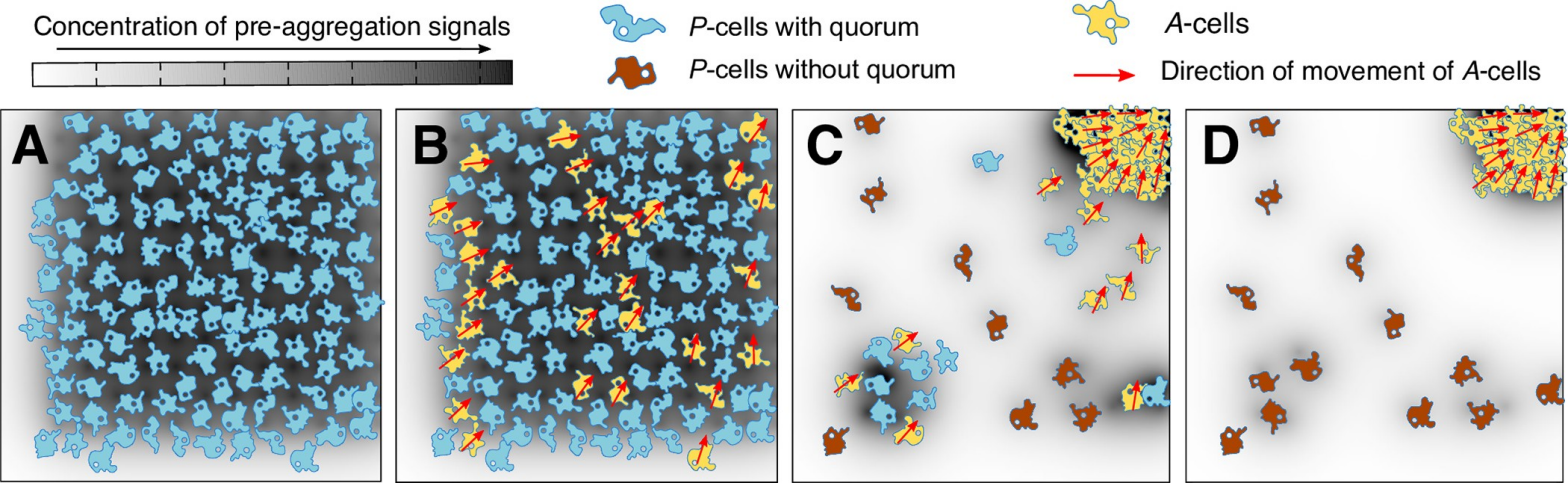
Developmental model schematic. (A) At high initial densities, all *P*-cells have a quorum to initiate aggregation. (B) With a strain-specific probability, some *P*-cells transition into *A*-cells. (C) As *A*-cells aggregate, some of the *P*-cells that did not transition into *A*-cells are left without a quorum. These are the loners. (D) At the end of development, *P*-cells far from the aggregate location are more likely to have been left without a quorum and to stay as loners.

Despite the many simplifications and consistent with the expectations above, this reduced self-organization model is powerful enough to qualitatively recapitulate most properties of the observed population partitioning (Fig 1F and S5A–S5C Fig), with the notable exception of the strain-specific variability in loner density, the characterization of which constitutes an interesting direction of future research. Importantly, the model recovers the plateau in the loner counts as a function of initially plated cell density (Fig 1F, S5A, and S5B Fig), and it suggests a possibility for the identity of loners in *D*. *discoideum* (Fig 2): they could be *P*-cells that did not make the probabilistic transition to the *A*-state when they had a quorum and that are left without a quorum when enough of their neighbors underwent the *P*-to-*A* transition and moved towards the aggregation center. The total loner density then depends on how quickly *P*-cells switch to the *A*-state relative to how quickly they are left without a quorum (*λ*/*ν*) and on how easy it is to maintain a quorum (see S5A and S5B Fig). Thus, the larger the *P*-to-*A* transition rate *λ*, the fewer loner cells left behind because *P*-cells sensing a quorum switch more synchronously to the *A*-state; conversely, the larger the aggregation speed *ν*, the more loners left behind because *A*-cells move away faster and narrow the time window in which *P*-cells maintain quorum (S5A Fig). Consequently, the farther a cell is from the aggregation center, the sooner it is left without a quorum, and the more likely it is to become a loner (S5C Fig), which is consistent with our experimentally observed spatial distribution (Fig 1C and S1 Fig). These results are derived from the numerical simulations of the full, spatially explicit model described above and are supported by the analytical solution of a mean-field, spatially implicit approximation that neglects the spatial effects of finite signal diffusion (*D* → ∞) but still accounts for cell movement with finite velocity (see Box 1 for a summary of analytical results and Section 2 of the S1 Text for details).

**Box 1**

In the spatially implicit limit (*D* → ∞, *ν* finite) and in the limit of large initial population size (*N*_0_ → ∞), the density of loners *ρ*_*L*_ can be calculated analytically, which reveals the interactions among model parameters (see S1 Text for detailed calculations). We find

$$\rho_{L}\propto \left\{ \begin{array}{l} \kappa (1-\frac{\lambda }{\tilde{v}}),\;if\lambda <\tilde{v}\\ \;0,\;otherwise \end{array},\right.$$

where *κ* = *θ*/*γ* is the ratio between the sensitivity threshold and the signaling rate, *λ* is the *P*-to-*A* transition rate, and $\tilde{v}$ is the velocity rescaled by the mean distance traveled by cells before joining the aggregate. This reveals a phase separation determined by the relative rates of the 2 transitions, *P* to *A* and *A* to *M* (Section 2.3 of S1 Text): the lack of synchronization introduced by the stochastic response to quorum only results in loners if the *A*-to-*M* transition is fast enough to leave cells with delayed *P*-to-*A* conversion without quorum.

These findings suggest that the *D*. *discoideum* loners could result from the interplay between the degree of synchronization in the *P*-to-*A* transition—determined by the strain-specific transition rate *λ*—and the amount of time that a quorum is maintained before *A*-cells join the multicellular phase—determined by the strain-specific cell velocity, *v*. However, other spatial components of the aggregation process can further shape the population partitioning, for example, signal diffusion, which determines the distance at which cells sense each other. Because the spatially implicit approximation neglects the role of finite diffusion, we investigate this possibility only via numerical simulations of the full, spatially explicit dynamics. Lower diffusivities result in higher loner densities (S5D and S5E Fig) because the signal remains highly concentrated around the emitters, and cells need to be more densely packed to maintain a quorum (S5F and S5G Fig). Moreover, decreasing the diffusivity differentially affects worse and better aggregators (Fig 1G, S5D and S5E Fig) because diffusivity and signal spreading are nonlinearly related (see Materials and Methods). These results mirror the experimentally determined dependence of loner densities on agar concentration, suggesting signal diffusivity as one (among possibly many; for example, cell velocity, attachment to substrate) potential mediator of this dependency. If diffusion is indeed a mediator, then, because loner densities responded to agar-concentration changes in a range that should not impede the diffusion of cAMP [35,36], these results further suggest that at least one of the molecules involved in the quorum-dependent transition should be large—for example, CMF [26], prestarvation factor (PSF), counting factor [37,38], or phosphodiesterase [39].

**Fig 3:**
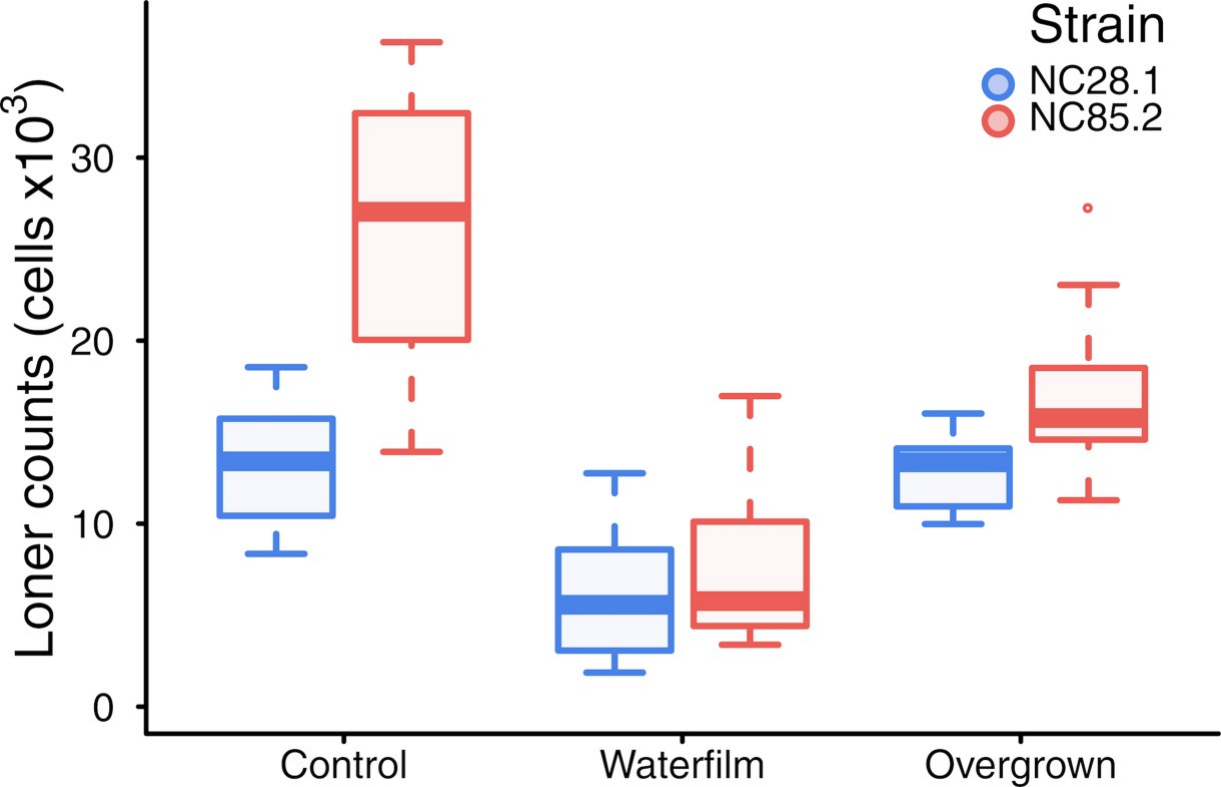
Well-mixed signaling changes loner behavior. Adding a thin film of water that dries after 4 hours (before aggregation begins) decreases the number of loners for both strains (*p* < 0.000001). Furthermore, allowing cells to exhaust their resources in suspension does not significantly change the behavior of the better aggregator (strain NC28.1) (*p* = 0.7), but it reduces the mean and variance of the number of loners of the worse aggregator (strain NC85.2) (*p* = 0.0006). The control is an independent replicate of the experiments in the framed portion of Fig 1E. Boxes, interquartile ranges; horizontal lines, medians; whiskers, 1.5× interquartile range from the median; points, outliers.

Notably, PSF and CMF are secreted during the growth phase and early starvation, respectively. Thus, the hypothetical above led us to investigate the potential role that these earlier signaling stages might play in regulating loner behavior. To test the hypothesis that early signaling regulates loner densities via diffusion, we proposed 2 independent experiments. First, we grew and plated cells as in the experiments from Fig 1, but upon plating the cells, we added a thin water film above them. The water film dried up in less than 4 hours (i.e., before the onset of aggregation) and therefore did not directly impact the aggregation dynamics. However, while the water film was present, it could facilitate the diffusion of signaling molecules. If limited diffusion of molecules secreted during these first 4 hours of starvation (such as PSF and CMF) promotes loner cells, then we would expect that the water film treatment would reduce the number of loners observed, which was indeed the case for both strains tested (Fig 3). Second, we let cells grow in bacterial suspension until resources were depleted and only subsequently plated them in agar gels. Under this treatment, the initial responses to resource depletion occurred in a well-mixed environment (i.e., at very high signal diffusion), and any signaling molecules secreted in this phase should have homogeneously reached all cells, thereby increasing their behavioral coordination. Accordingly, we expected cells plated after being overgrown to leave behind fewer loner cells, which was indeed the case for the worse-aggregating strain (Fig 3). Altogether, although we cannot exclude the possibility that different factors may be responsible for each of the 2 experiments—for example, mechanical effects of the water film for the former and cell cycle synchronization for the latter—the fact that diffusion can account for the results of both of these independent experiments strongly suggests that our hypothesis is true. Importantly, these experiments support the proposal that vegetative or early starvation signaling—and not the later cAMP relay signaling and synchronization, as previously inferred using knockouts [10]—could be a critical stage at which loner behavior is regulated and the natural variation that we observed is produced.

Interestingly, both experiments revealed a convergence in the loner behavior of the 2 strains, i.e., the magnitude of the decrease in the loners of the worse aggregator was higher than that in the loners of the better aggregator (Fig 3). These empirical results mirrored our theoretical predictions for increasing the diffusivity of signaling molecules: specifically, theoretically we predicted that the high diffusion environments (such as those obtained by adding a water film or by keeping cells in a well-mixed suspension) should not only decrease the loner number of any given strain but also cause the differences between strains to decrease (Fig 1G). This close agreement with the theoretical predictions further reinforces our inferences regarding the importance of diffusion.

### The aggregator–loner partitioning depends on the identity of neighboring cells, i.e., coaggregating strains affect each other’s aggregation performance

**Fig 4:**
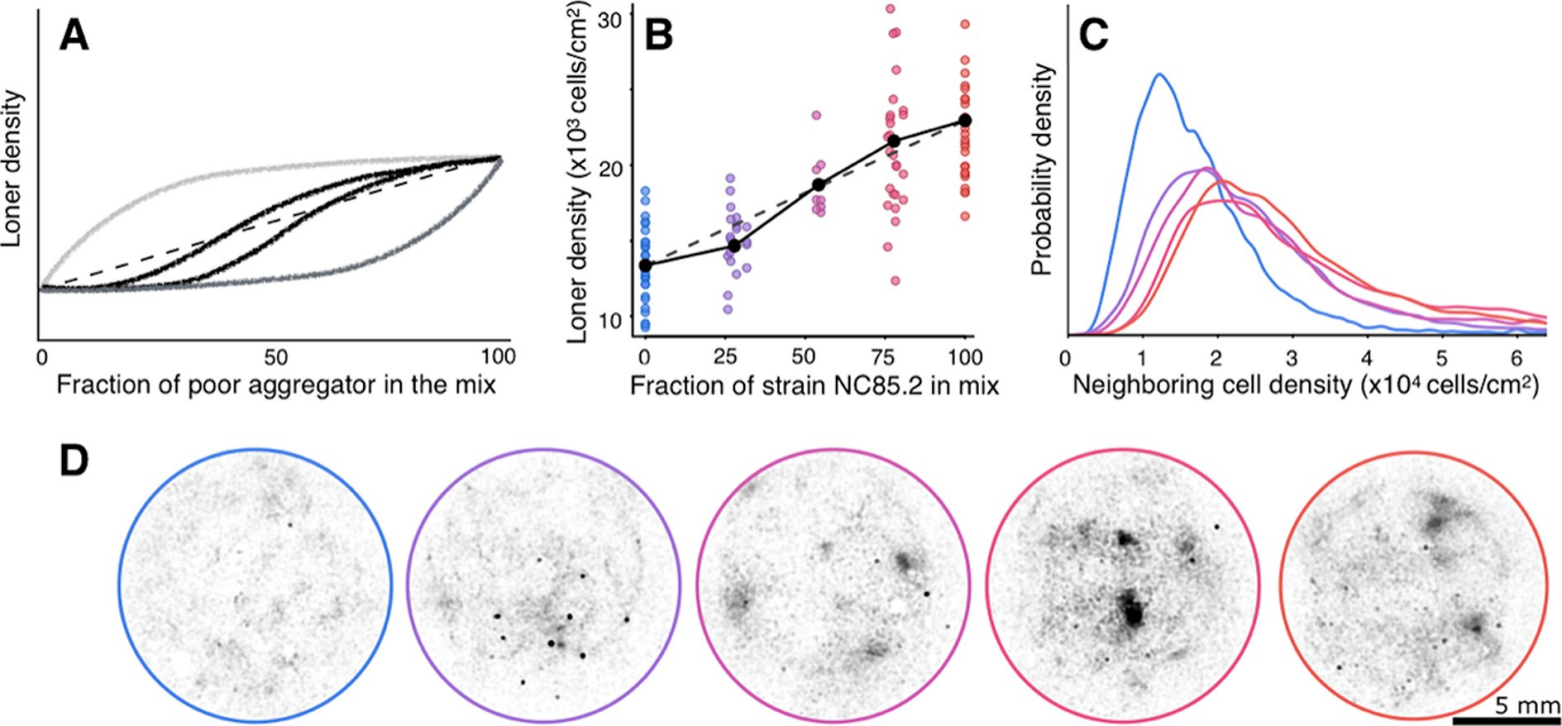
Co-occurring strains interact during development. (A) Schematic of the range of theoretically predicted loner densities at different mix proportions of 2 co-occurring strains (thick curves). Dashed line, expected loner densities if cells commit to aggregation independent of the identity of their neighbors. (B) Experimentally observed loner densities for different mix proportions of a better (NC28.1, blue) and a worse (NC85.2, red) aggregating strain in 3% agar. Black points, mean loner densities for each of the 5 proportions. Dashed line, same as in (A). Solid lines, piecewise linear regressions, which deviate significantly from the linear fit (*p* = 0.019; see Materials and Methods). The significant departure from the linear fit is reinforced by bootstrapping and maximum likelihood analyses (see S7 Fig and Materials and Methods). The endpoints (0% and 100%) represent an independent replicate of the experiments in the framed portion of Fig 1E. (C) Experimentally observed spatial patterns of each mix proportion are characterized by the local cell density around each cell (see Materials and Methods). Narrower distributions (NC28.1, blue curve) correspond to more homogeneously distributed loners. Broader distributions (NC85.2, red curve) correspond to more clumped loners. (D) Experimentally obtained loner position maps are shown for each of the mixed proportions. Colors in (C) and (D) correspond to colors in (B).

Collectively, the results above show that the population partitioning stems from interactions between genotype and environment and suggest that cell signaling mediates these interactions. This suggestion raises the possibility that a strain’s partitioning could also be influenced by the presence of other strains via cross-signaling. If a cell’s commitment to aggregation were independent of the identity of co-occurring strains, a mix of strains would leave behind a total mixed-loner density that is the linear combination of the 2 strains’ loner densities (see Materials and Methods). Our model, however, predicts developmental interactions between co-occurring strains that produce a diversity of departures from linearity depending on signaling parameters (Fig 4A and S6 Fig).

When we plated well-mixed cells of the strains NC28.1 (better aggregator) and NC85.2 (worse aggregator) at different frequencies and left them to codevelop under starvation conditions, we found agreement with one of the possible theoretical predictions. The total loner density of the mixed strains deviated significantly from the linear combination, mapping out a sigmoidal curve (Fig 4B and S7 Fig), which was one of 3 outcomes we encountered in the simulations. Thus, when the better aggregator was more abundant in the mix (25%:75%), there were fewer total loners than predicted by the linear combination; conversely, when the worse aggregator was more abundant (75%:25%), there were more total loners. That strains influence each other’s partitioning is consistent with existing results using knockouts [10], and it is particularly interesting in light of prior work showing that genetically heterogeneous multicellular aggregates occur naturally [40], thus allowing for potential interactions between strains that can alter each other’s life-history investments [14,16]. Whether or not such interactions occur within the aggregate [11,17,41], our results reveal that they do occur earlier in the developmental process.

Our experimental setup does not allow us to determine which strain each loner cell belongs to, and therefore, we cannot directly infer how the developmental interactions affect each strain. We can nevertheless explore this question theoretically. The model has too many parameters to evaluate its behavior in the entire parameter space. However, both extensive simulations and analytical calculations that covered large regions of the parameter space (see Section 2.4 of S1 Text for details) revealed that, when the theoretical density of the mixed-strain loners had a sigmoidal shape similar to the experimental one, the loners of the better aggregator went to zero as the mix frequency of the worse aggregator increased (S8A and S8B Fig). This occurred because the more sluggish loners of the worse aggregator maintained a quorum long enough for the better aggregator to aggregate perfectly. Hence, the better aggregator became even better in the presence of the worse aggregator, which, in turn, became even worse, and this enhanced the difference between the 2 strains. Experimentally, the spatial distribution of the mixed-strain loners provides insight into their potential composition (Fig 4C and 4D): as soon as the worse aggregator is part of the mix (even at the lowest frequency), the spatial distribution of the mixed loners is almost identical to that of the worse aggregator—and strikingly different from that of the better aggregator—suggesting that, indeed, the mixed-strain loners predominantly comprise the worse aggregator. Importantly, developmental interactions between co-occurring strains do indeed have consequences for the life-history investments of individual strains.

Although not exhaustive, additional simulations and analytical calculations also revealed a complementary outcome of developmental interactions: a better aggregator can become worse in the presence of a worse aggregator, while the worse aggregator becomes better, which diminishes the difference in partitioning behavior between the 2 strains (see S8A and S8C Fig and S1 Text).

### Coaggregation may foster slime-mold diversity across spatiotemporal scales

Such developmental interactions that alter life-history investments could severely impact strain fitness, alter *D*. *discoideum* diversity, and threaten the persistence of social behavior [14]. It is therefore crucial to understand their consequences. Here, we theoretically explore potential long-term ecological consequences of strains coaggregating and codeveloping, which also provides some insight into potential consequences of the aggregator–loner partitioning behavior for strain fitness.

**Fig 5:**
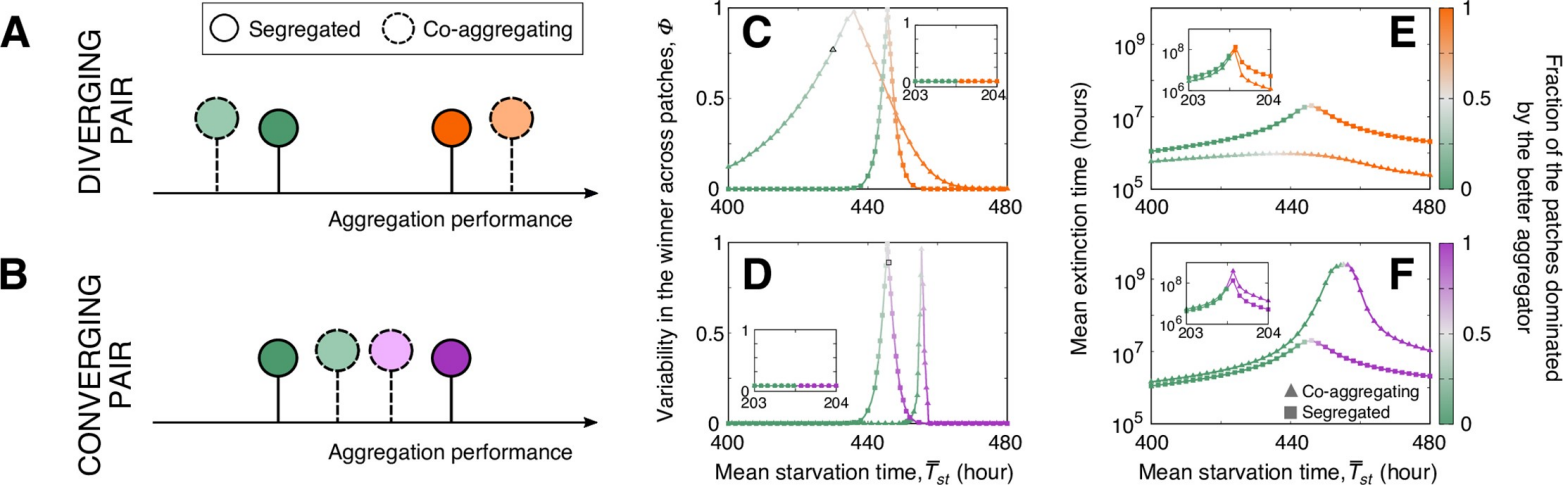
Ecological implications of developmental interactions. (A, B) Codevelopment makes strains (different colors) diverge (A) or converge (B) in their partitioning behavior. (C–F) Effect of coaggregation versus segregated development on (C, D) the identity of the winner (Eq 6 in Materials and Methods) and (E, F) extinction times as a function of environmental harshness (longer for larger ${\bar{T}}_{st}$). Panels (C, E) and (D, F) are predictions for the pairs of strains in S8A and S8C Fig, respectively. Green strain is the same across all panels. Main panels, stochastic environments; insets, deterministic environments (see Materials and Methods). Color gradients indicate the change in the fraction of patches dominated by each of the mixed strains. Parameterization as in S8A and S8C Fig.

In nature, when 2 strains co-occur, they are likely to coexperience not just one starvation event (as above) but several growth–starvation cycles, and thus, they are likely to repeatedly coaggregate. Because the 2 outcomes of coaggregation predicted by our population-partitioning model are reminiscent of 2 classical evolutionary routes to diversity maintenance—character displacement (strains become more different; Fig 5A) and quasineutrality (strains become more similar; Fig 5B)—they are likely to have consequences for slime-mold diversity in the long term.

Therefore, it makes sense to investigate the potential long-term ecological (biodiversity) consequences of repeated coaggregation. To do so, we embedded our population-partitioning model into an ecological framework of pairwise strain competition for resources over successive growth–starvation cycles [11,12,41] (S9 Fig; see Materials and Methods). Recent empirical and theoretical work has revealed several life-history traits that are likely to have ecological consequences, such as division rates, spore germination success, cell size, and the existence of loners [10–12,17,41,42]. Here, we aim to investigate only whether this newly proposed mechanism of loner allocation and its consequent developmental interactions could have an ecological impact and should therefore be included into ecological frameworks that explore *D*. *discoideum* diversity. Therefore, we assume strains to differ only in their partitioning behavior, and we keep all other traits constant. Moreover, we assume that strains are neutral with respect to their spore/stalk allocation pattern, which is also independent of aggregate size.

Whereas in previous sections a starvation event referred exclusively to the period of aggregation following resource depletion (and thus ended when all aggregating cells joined the aggregate), in this long-term analysis, a starvation period is the time between 2 growth periods (i.e., the time from when resources are depleted until they replenish). The environment is characterized by the distribution of starvation-period durations (i.e., resource replenishment times). In this framework, we assume that during starvation periods, spore mortality is negligible, while starving loner cells progressively die according to empirically measured survival curves [15]. Accordingly, although loner cells have been shown to remain viable for many days [10,11], for long enough starvation periods, no loner cell survives (S9 Fig; see Materials and Methods). When resources replenish, surviving loner cells begin to divide immediately, while spores take a certain amount of time to germinate and only subsequently divide (i.e., spores have a reproductive delay relative to surviving loners). Since resource replenishment times in *D*. *discoideum*’s natural environments are not known, we explore a range of environments, from very harsh (conditions under which most or all loner cells die during most of the starvation periods) to very mild (conditions under which most loner cells survive most of the starvation periods). Furthermore, we consider both deterministic environments, in which all starvation periods are of equal size, and stochastic environments with exponentially distributed starvation periods (which are thus characterized by the mean replenishment time).

We ran pairwise competition simulations for the 2 pairs of strains in S8 Fig, which belong to the 2 different developmental outcomes discussed in the previous section. Henceforth, we will refer to the pairs as the diverging (S8A and S8B Fig) and the converging (S8C and S8D Fig) pair, respectively. For each pair of strains, we compared the outcome of the 2 strains mixing and coaggregating to that of a hypothetical scenario in which the 2 strains perfectly segregated and avoided coaggregation. Between 2 starvation periods, the differential survival of spores and loners, coupled to the spore delay in consuming the returning resources, led to changes in the relative abundances of the strains (i.e., a difference in strain fitness). Regardless of whether coaggregation occurred within any environment, there was competitive exclusion. Therefore, the outcome of the competition was characterized via the identity of the winner and the time it takes for the loser to become extinct. In each of the 2 simulated pairs, we found that the strain that leaves behind more (fewer) loners is more competitive, and thus has a higher probability of being the winner, in environments with shorter (longer) mean replenishment times, consistent with previous work [11]. In deterministic environments, the identity of the winner was also deterministic and not altered by coaggregation (inset of Fig 5C and 5D); however, coaggregation did alter the time to extinction of the loser (inset of Fig 5E and 5F). On the contrary, in stochastic environments, there is a range of environments where the identity of the winner is uncertain, and that range is drastically altered by coaggregation (Fig 5C and 5D). As in deterministic environments, coaggregation also influenced the time to extinction of the loser (Fig 5E and 5F).

These results suggest that shifts in strain partitioning behavior due to coaggregation could play a role in how strain–strain competition for resources is mediated. Notably, this is true even in the harshest environments, where no loners (of either strain) ever survive the starvation periods and therefore loners do not contribute to subsequent growth (i.e., have no direct contribution to fitness). This is because, because of developmental interactions, even the transient existence of the loners can indirectly impact fitness by impacting the spore allocation strategies (and, therefore, the fitness) of coaggregating strains. Specifically, in such harsh environments, there is no uncertainty in the identity of the winner: when pairs of strains compete for resources across many growth–starvation cycles, the worse aggregator (i.e., the strain that leaves more loners behind) always goes extinct. Nevertheless, because of strains shifting their partitioning behavior, the time to extinction of the worse aggregator changes. For the diverging pair of strains, coaggregation causes the worse aggregator to become worse and thus produce even fewer spores (S10A Fig) while causing the better aggregator to become even better, which leads to shorter time to extinction of the worse aggregator (S10B Fig). For the converging pair, coaggregation causes the worse aggregator to become better and thus increase its spore production (S10C Fig), which leads to increased representation in the subsequent growth cycle and therefore to longer time to extinction of the worse aggregator (S10D Fig).

**Fig 6:**
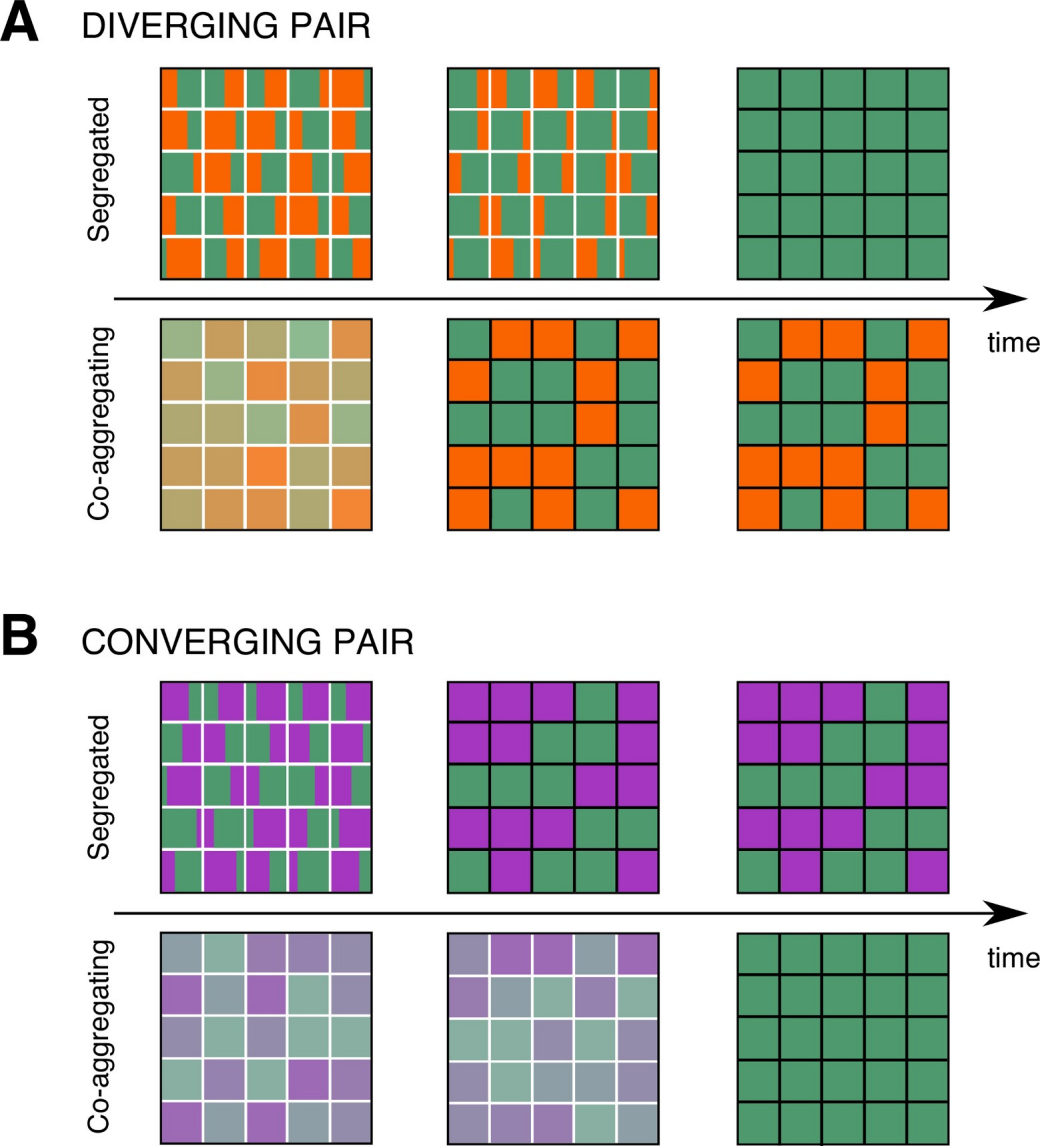
Model results of the effects of developmental interactions on transient alpha diversity and stationary beta diversity in an environment discretized into 25 patches (see S9 Fig). (A, B) White interior boundaries symbolize a transient patch-level population composition; black interior boundaries symbolize a stationary patch-level population composition. (A) Strains diverge in their partitioning behavior because of developmental interactions during coaggregation. In a stochastic environment with mean starvation time ${\bar{T}}_{st}$ = 430 hours (symbol with black boundary in Fig 5E), the mean extinction time decreases (decreased transient alpha diversity), but the variability in the winner across patches increases compared to segregated development (increased stationary beta diversity). (B) Strains converge in their partitioning behavior because of developmental interactions during coaggregation. In a stochastic environment with mean starvation time ${\bar{T}}_{st}$ = 448 hours (symbol with black boundary in Fig 5E), coaggregation increases transient coexistence (and transient alpha diversity) relative to segregation. However, it also eliminates the variability in the winner and thus stationary beta diversity. In both (A) and (B), snapshots are taken for the initial condition (left column), at an intermediate time at which scenarios with lower alpha diversity have reached the stationary state (central column), and when both coaggregation and segregated development have reached stationarity (right column).

Finally, we attempt to untangle how these potential diversity effects of coaggregation play out at different spatial scales. To capture the broadest effects of both uncertainty in the winner and modified time to extinction of the loser, we focused on mild-to-medium environments. To explore the different spatial scales, we discretized the environment into small-scale patches with identical replenishment conditions. Competition between pairs of strains occurred within each patch, and there was no dispersal between patches. Each patch was initialized with the 2 strains in random proportions (see Materials and Methods, S9 Fig). Importantly, this setup allows us to investigate the effects of coaggregation on alpha (intrapatch) and beta (interpatch) diversity, but it does not introduce any intrinsic spatial heterogeneity because all patches have the same environmental conditions. As expected, within each patch, we found competitive exclusion. However, at the level of the landscape, if the replenishment conditions are within the range in which the identity of the winner is uncertain, there can be coexistence. The 2 pairs had distinct biodiversity signatures. Specifically, the shorter times to extinction characteristic of the diverging pair resulted in lower transient alpha diversity compared to the segregated model. However, their diverging interaction also broadened the environmental range in which competition leads to nondeterministic exclusion, resulting in higher stationary beta diversity compared to the segregated model (Fig 6A). The converging pair yielded the opposite outcome for both alpha and beta diversity (Fig 6B).

## Conclusion

Here, we showed natural variation and heritability in the aggregator–loner partitioning behavior of naturally co-occurring strains of *D*. *discoideum*. Strikingly, the seemingly asocial loners are not a separate, independently determined subset of cells, but rather, they appear to arise dynamically from the collective process. Coupling experiments and theory, we proposed that the aggregator–loner partitioning behavior is governed by a stochastic cell-level decision-making process (i.e., a process yielding a behavioral output given an input) mediated by cell signaling and modulated by both the abiotic and the biotic context. Importantly, the loner–aggregator partitioning resulting from the decision-making process encoded by our model does not a priori maximize the fitness of any given strain, i.e., being a loner is not a priori the result of “strategic decision making” [43,44]. These investigations of collective behavior point to the existence of previously unknown stochastic aspects of *D*. *discoideum* development. Finally, we used a theoretical approach to explore the ecological consequences of these findings and concluded that the codevelopment of different strains can impact diversity across multiple scales. These results, arising solely from interactions between ecology and development, recapitulate the biodiversity outcomes of classical eco-evolutionary interactions. Overall, our results highlight the necessity of an integrated approach to collective behaviors, including multicellularity: studying asocial life-history strategies could provide insights into collective behavior and development, and studying development could provide insights into ecological dynamics and parallels with well-studied eco-evolutionary processes.

Although here, we focused on the ecological consequences of the loner–aggregator partitioning, our findings suggest avenues for future research into the evolution of aggregative multicellularity and social behaviors. Many factors could undermine the integrity of social complexes, such as free-riding, whereby individuals reap the benefits of group living without paying the costs. In slime molds, free riders—strains that never contribute to stalk formation in mixes—have been found both in the wild [45] and in the lab [46]. If under selection, these heritable loners, invulnerable to the threats to the multicellular stage but capable of reachieving multicellularity via their offspring, could constitute insurance against such threats and could therefore be critical to the evolution and persistence of aggregative multicellularity. This hypothesis is consistent with the handful of theoretical studies on cooperative behaviors that have considered social loners [47,48].

Finally, beyond multicellularity and sociality, our results have potential implications for the broadly analogous loner behaviors identified across a variety of systems in which some form of coordination or synchronization is observed, from insects [4] to vertebrates [2,5] to plants [3]. Our findings represent the first demonstration that loner behaviors can indeed exhibit natural variation and heritability, which can have significant ecological consequences. As such, our work motivates a broader investigation into loner behaviors in other biological systems. While the mechanisms underlying the existence of loners are likely different across systems, the widespread existence of loners and the possibility that they could in fact be shaped by selection suggest an interesting conjecture: that, in general, imperfect coordination among individuals may enable evolution to shape population-partitioning strategies in ways that could be instrumental for behavioral diversity and for the persistence of the collective stage and thus for system-level robustness.

## Materials and methods

### Experiments

*D*. *discoideum growth and plating*. NC28.1, NC34.1, and NC85.2—three clonal lineages of *D*. *discoideum* originally isolated from Little Butt's Gap, North Carolina [49]—were obtained from dictyBase [50] and grown on *Klebsiella aerogenes* lawns prepared on SM agar plates [51]. After the *D*. *discoideum* cells aggregated and formed fruiting bodies, spores were harvested and used to inoculate 3 mL of a *K*. *aerogenes* suspension in SorMC buffer (OD600 of 8). The suspension was kept in a shaker for 24 hours and then used to inoculate 12 mL of the *K*. *aerogenes* suspension. During growth, *D*. *discoideum* cell densities were kept below 3 × 10^6^ cells/mL. After 24 more hours, when cell densities (of all strains) were between 1.5 × 10^6^ and 3 × 10^6^ cells/mL, the suspension was cooled to 4°C for 5 minutes in preparation for the homogeneous starvation of the cells. The suspension was then centrifuged at 700 × *g* for 3 minutes at 4°C, and the remaining pellet was resuspended in 10 mL of SorMC buffer. The spinning and resuspension cycles were repeated 3 times to wash away any remaining *K*. *aerogenes* cells [50]. For the final resuspension, the cell concentration was 10^7^ cells/mL.

From each stock suspension, a dilution series in SorMC buffer (80%, 70%, …, 20%) was obtained. In addition, a 5% dilution was prepared from each stock suspension. The 5% dilutions were below the critical aggregation density, and they were used to estimate the total amount of cells in the other samples coming from the same stock suspension. Cells were plated on non-nutrient agar gels (2%, 3%, and 4% concentrations) cast in 1.5-mm acrylamide gel casts (Bio-Rad, Hercules, CA, USA). Each of the diluted and undiluted cell suspensions was applied to the agar substrates as a 10 μL droplet.

The samples were then left to develop in a moist dark chamber at 21°C until the streaming phase of aggregation was over and most of the aggregates were already at the slug stage or starting to form fruiting bodies. Because development times depended on strain and agar concentration, the time to reach slug stage was different for each experiment. Experiments usually reached the slug stage between 14 and 20 hours (and never took longer than 22 hours). If left to further develop in petri dishes, slugs migrate over what was once the aggregation territory, potentially picking up some stray loner cells and shedding others, and the extent of this migration depends on a variety of experimental conditions (for example, luminosity, temperature, air flow). However, in natural substrates such as soil and dung, cells aggregate right below the surface, and it is believed that slugs use light and air circulation cues to move directly towards the surface of the substrate, away from where aggregation occurred [52,53]. In order to minimize counting as loners the cells shed by migrating slugs and to minimize missing loners picked up by passing slugs, when all aggregates entered the slug stage or were beginning to develop into fruiting bodies, we halted development by lowering the chamber temperature to 4°C. Even though the 5% diluted suspension samples never aggregated, they were also left in the chamber for the full length of the experiment. This circumvents the problem of residual divisions post-resource removal. The diluted and nonaggregated samples were then used to estimate the total number of cells in the undiluted aggregated samples.

In order to check for the consistency of the dilution procedure, 5%, 10%, and 15% dilutions of NC34.1 strain stock solution were also prepared and plated on 3% agar substrates. A linear Gaussian model with intercept 0 was fitted to the cell counts of different dilutions of the same suspension. The standard deviation of this model was taken to be the error intrinsic to the dilution process (S3A Fig).

For mixed-strain experiments, strains NC28.1 and NC85.2 were used. They were grown, washed, and resuspended separately. Then, without diluting the suspensions, different mixes were made (25%, 50%, and 75% of the initial strain NC28.1 suspension). A 5% dilution was made for each of the 2 pure strain suspensions. Each of the mixes, pure strain suspensions, and dilutions were plated as 4 replicates for each experiment. These 5% dilutions were used to estimate the proportions of cells of each strain in each of the experiments. We used 3% agar substrates.

For the resource-depletion experiments (Fig 3), cells of strains NC28.1 and NC85.2 were grown in bacterial suspensions until they reached either a density of 1 × 10^6^ cells per mL (conditions under which bacteria were still plentiful) or for 10 hours longer than that (conditions under which bacteria have just been depleted). In both treatments, cells were then washed from bacterial leftovers and left to aggregate on 3% agar gels.

For the water film experiments (Fig 3), cells of strains NC28.1 and NC85.2 were grown in bacterial suspensions until they reached either a density of 1 × 10^6^ cells per mL (conditions under which bacteria were still plentiful), washed as previously described, and plated on 3% agar gels. An ultrasonic atomizer (from CVS) was used to uniformly apply a thin layer of water (approximately 100 microns) over the gels. The water film dried in less than 4 hours.

*Imaging samples and counting cells*. An ultrasonic atomizer (from CVS) was used to uniformly apply a 0.5-mm–thick layer of warm imaging solution (SorMC containing 0.3 mol/L of dextrose and 0.05% agar) to the samples. After resting for 10 minutes at room temperature, cells assume a spherical shape and detach from neighboring cells. Samples were then photographed with a Canon t5i DSLR camera (Melville, NY, USA) on a Nikon Ti-Eclipse inverted microscope (Nikon, Tokyo, Japan) equipped with a 10× objective. The imaged area, a square of side 1.5 cm, was large enough to encompass the initial cell plated area plus a buffer zone that ensures that all cells were imaged. Custom software using the OpenCV package for Python was then used to count the cells that did not join aggregation centers.

The error of automatic cell counts was estimated by taking samples of images of cells in various densities, manually and automatically counting them, and fitting a linear Gaussian model with slope 1 and intercept 0 (S3B Fig). The standard deviation of the fitted model was taken as the automatic counting error.

Total plated cell counts in each sample were estimated by counting the nonaggregated 5% dilution of the corresponding stock suspension and multiplying by the dilution factor of that particular sample. We do not assess differential loner viability, which might effectively increase the difference in loner allocation between strains.

*Loner map construction*. After the position of each loner cell was determined from the composite images, a representation of the entire experimental area was created by plotting at the position of each loner cell a semitransparent black circle of 200 μm diameter (about 20 times the diameter of a cell) (Figs 1C, 4D and S2A Fig). When loners are dense (i.e., loner cells have many close neighbors), the circles representing those cells overlap, and the region appears black; when loners are sparse, circles are less likely to overlap, and the region appears light gray.

*Video acquisition and analysis*. Time lapses were acquired (a picture every 5 minutes) using the same imaging apparatus previously described but with a 4× objective (S1–S6 Videos). Videos were then used to estimate loner coverage as a function of distance from the aggregation center. For each aggregation territory in a video, we identify the frame in which slug formation is completed (one frame before the slug “falls” on the aggregation territory). ImageJ was then used to threshold the area within the territories covered in loner cells. The territories were broken in concentric annuli (with maximum diameter of 1 mm; value chosen from independent measurements of territory sizes), and the loner coverage proportion was plotted as a function of the distance of the ring to the aggregation center (S1 Fig).

*Spatial pattern analysis*. For each cell in each experiment, the local cell density was calculated in a neighborhood with a radius of 10% of the total experimental radius. Cells that were not within the center of the experimental area (defined by a radius of 60% of the total experimental radius) were not included in the analysis to avoid border effects. For each strain and agar concentration, neighborhood densities of cells of all experiments were pooled together in a probability distribution. The results were qualitatively similar for other neighborhood radii, but because the imaging treatment might shift the position of the cells a bit, we chose a radius that is considerably larger than this effect.

*Statistical analyses of mixed-strain experiments*. Given a constant initial cell density, we tested whether the loner–aggregator partitioning process of a strain is influenced by the genotypic identity of its neighbors. We let *P*_*i*_ be the proportion of cells of strain *i* that stay as loners. We determined that this depends on *ρ*_0_, the initial density of the population, such that the density of loners in a clonal population of strain *i* at density *ρ*_0_ is $\rho_{L}^{i}=\rho_{0}P_{i}(\rho_{0})$. Here, we investigated whether in mixes *P*_*i*_ is also a function of the fraction Π_*j*_ = 1 – Π_*i*_ of cells of co-occurring strain *j* (where Π_*i*_ is the fraction of cells of strain *i*). The total loner density in the mix can be expressed as $\rho_{L}^{\mathrm{mix}}={\Pi_{i}\rho }_{0}P_{i}(\rho_{0},\Pi_{j})+{\Pi_{j}\rho }_{0}P_{j}(\rho_{0},\Pi_{i})$, where *ρ*_0_ is the initial total density of plated cells and Π_*i*_ is the fraction of strain *i* in the initial mix.

Null hypothesis. If *P*_*i*_ is not a function of Π_*j*_, then $\rho_{L}^{\mathrm{mix}}=\Pi_{i}\rho_{L}^{i}+\Pi_{j}\rho_{L}^{j}$, which is the linear combination of the expected loner counts for each of the strains composing the mix (null hypothesis).

Statistical test 1. Piecewise linear regression (Fig 4). We measure departures from this linear expectation by fitting a piecewise linear regression to the data. The *p*-value shows how often data drawn from the global linear fit generate piecewise linear regressions with more extreme inclinations.

Statistical test 2. Maximum likelihood (S7A–S7C Fig). We also use a maximum-likelihood–based model selection to test for nonlinearity. We let $\rho_{L}^{\mathrm{mix}}=\rho_{L}^{i}+f(\Pi_{j})(\rho_{L}^{j}-\rho_{L}^{i})+\epsilon$, where *f* is the function of interest and *ϵ* is a normally distributed noise term. If *f*(Π_*j*_) = Π_*j*_, then we recover the null hypothesis. In addition, for *f* we explore 3 other functional forms: sigmoidal, convex, and concave, which are given by a shape parameter *a* as follows:

$$f(\Pi_{j})=\frac{a\Pi_{j}}{1+(a-1)\Pi_{j}}\;-\;convex\;if\;0<a<1;\;concave\;if\;a>1,$$

$$f(\Pi_{j})=\frac{I_{a}(\Pi_{j})-I_{a}(0)}{I_{a}(1)-I_{a}(0)}\;where\;I_{a}(x)=\frac{1}{1+e^{-a(x-0.5)}}\;-\;sigmoidal.$$

We also considered 3 forms for the noise term: a homoscedastic structure, a constant coefficient of variation, and a heteroscedastic structure, given respectively by

ϵ∼N(0,c),

$$\epsilon ∼N\left(0,\frac{c}{\rho_{L}^{i}+f(\Pi_{j})(\rho_{L}^{j}-\rho_{L}^{i})}\right),\;and$$

$$\epsilon ∼N(0,c_{i}\Pi_{i}+c_{j}\Pi_{j}).$$

We computed the ΔAIC, the difference in AIC (Akaike Information Criterion) between a given model and the best model in the candidate set. Credible intervals were built for the shape parameter *a* using log–likelihood ratios.

Statistical test 3. Bootstrapping analysis (S7D and S7E Fig). For each of the 5 strain mix proportions, empirical distributions were bootstrapped, and 50,000 data sets were constructed. For each resampled data set, a linear regression was performed using only the pure strain experiments, and another linear regression was performed using only the mixed-strain experiments. The difference between these inclinations is a measure of the nonlinearity of the data set.

### Theory

*Population-partitioning model*. We implement the spatially explicit developmental model on a square system of lateral length *ℓ* = 0.2 cm that represents a single aggregation territory. Time is discretized in short intervals of length *dt* = 0.01 hour; our results are independent of the value of *dt*. Within each time step, the internal state and the position of every cell can be updated. Since reproduction and death are negligible over the temporal scales of aggregation, the total population size is conserved during each run of the model.

Consistent with the experimental setup, we initialize the simulations immediately after resource exhaustion with *N*_0_ discrete and randomly distributed cells assumed to be in a preaggregating state, *P*. Thus, the initial density of cells is *ρ*_0_ = *N*_0_/*ℓ*^2^. *P*-cells do not move; they emit signal at a constant strain-specific rate *γ* and sense it with a strain-specific sensitivity threshold *θ*. Within each time step *dt*, *P*-cells that sense a local signal density higher than the strain-specific sensitivity threshold *θ* may become aggregating *A*-cells with a strain-specific probability *λdt*. A detailed description of how signal density is obtained at the position of each cell is below.

*A*-cells move in the direction of the aggregation center, which is exogenously imposed in the center of the system, making a straight displacement of length *νdt* in every time step with cell velocity chosen according to experimental measures [54]. This movement pattern simplifies the complexities of *D*. *discoideum* motion during aggregation, such as the tortuosity in single-cell trajectories caused by imperfect chemotaxis [31]. *A*-cells stop sensing signal, but they continue to emit it at the same strain-specific rate, *γ*. When *A*-cells cross the location of the aggregation center (center of the system) in one of their displacements, they adhere to the mound and become multicellular *M*-cells. *M*-cells do not move and neither emit nor sense signal. Both the *A*-to-*P* and the *P*-to-*M* transitions between cell states are irreversible.

Simulations are allowed to run until the time between 2 consecutive cell arrivals to the mound is larger than a fixed value *t*_*arr*_ = 1 hour. Alternatively, we explore the effect of a fixed aggregation time by finalizing the simulations after an exogenously imposed time (results not shown). Neither qualitative nor quantitative differences were observed between these 2 ending conditions, provided that both of these times were sufficiently large. We compute the final density of loners by counting the number of cells that do not belong to the mound at the end of each model realization and dividing it by the area of the system, *ℓ*^2^. Because of the low skewness of the distribution of loner densities obtained from independent realizations, we use the mean loner density, which is obtained by averaging over 100 realizations. A summary of the model parameterization is provided in S1 Table.

Computation of signal density. Signal is released by both *A*- and *P*-cells, but it is sensed only by *P*-cells. The signal density, *σ*, at time *t* at the position ***r*** of a focal *P*-cell is

$$\sigma (r,t)=\sum_{r^{\prime }\neq r}\sigma_{r^{\prime }}(|r-r^{\prime }(t)|),$$ (1)

where the index of the sum runs over the locations of all other *A*- and *P*-cells in the system, ***r*** − ***r***′(*t*)| is the distance between the focal cell and these other cells, and *σ*_***r***′_ gives the individual contribution of a cell at location ***r***′ to the total signal density. Since *P*-cells do not move, ***r*** does not depend on *t*; similarly, ***r***′(*t*) is either constant (if it is the position of another *P*-cell) or not (if it is the position of an *A*-cell). Our assumptions that signal is continuously released by each cell at a strain-specific rate *γ*, diffuses in the system with diffusion constant *D*, and spontaneously decays at rate *η* lead to a stationary profile in which signal density decreases with the distance from the emitter (see Section 1 of the S1 Text for a detailed calculation of this profile),

$$\sigma_{r^{\prime }}(|r-r^{\prime }(t)|)=\frac{\gamma }{2\pi D}K_{0}\left(\sqrt{\frac{\eta }{D}}|r-r^{\prime }(t)|\right).$$ (2)

*K*_0_ is the zero-order modified Bessel function of the second kind, and we allow *D* to vary within a range of values that cover different experimental conditions [55–57]. Because in the experiments, aggregation occurs simultaneously on several adjacent aggregation territories, signals may diffuse from one territory to another. To allow for this possibility in the model, the distances between the sensing focal *P*-cell and each of the emitters are measured using periodic boundary conditions.

*Pairwise competition model to explore long-term ecological effects of coaggregation*. This model captures the competition of a pair of strains during a sequence of growth–starvation periods, with the population partitioning between loners and aggregators occurring at the onset of starvation. The expected length of the starvation periods (i.e., mean starvation time) defines the environmental conditions. We discretize each environment into # = 10^4^ isolated patches (no cell dispersal between them) of area 1 and identical environmental conditions. The model architecture broadly follows [41]; however, in order to isolate the ecological consequences of coaggregation solely due to the shifts in strain partitioning behavior, we assume that strains are identical in all life-history traits except in their partitioning behavior between loners and aggregators, which follows the outcome of the developmental model described above. We assume that the loner–aggregator partitioning curve is constant for all starvation events. Therefore, we do not consider mutation or horizontal gene transfer, which could alter strains’ aggregation behavior. Accounting for strain difference in other life-history traits and tradeoffs among them constitutes an important expansion towards a more complete understanding of the ecology of slime molds [12,17,41].

Growth. During growth, free-living amoebae of 2 different strains compete for a shared pulse of resource within each patch. The size of the resource pulse determines the environment’s carrying capacity, and it is fixed and large to guarantee that the population of cells in the patch is also large and that its fluctuations do not affect the aggregator–loner partitioning (i.e., aggregation occurs for population sizes that lie in the region in which loner density plateaus in S5 Fig). Mathematically, the growth dynamics is given by a Monod-like equation:

$${\dot{X}}_{i}=\frac{cR}{R+R_{1/2}}X_{i}(i=1,2),$$ (3A)

$$\dot{R}=\frac{-cR}{R+R_{1/2}}\sum_{i=1}^{2}X_{i},$$ (3B)

where the dot indicates the time derivative, *X*_*i*_ is the population size for strain *i*, *R* is the amount of resources, and *R*_1/2_ is the abundance of resources at which the growth rate is half of its maximum *c*. The initial frequency of each strain in the mix is drawn from a standard log-normal distribution, and the total initial population size *X*_0_ is normalized to the size of the resource pulse *R*_0_ so that the population starts close to the carrying capacity and simulations are faster. Our results are unaffected by considering different initial conditions. The growth phase finishes when resources are below a starvation threshold (R* = 1) that is exogenously imposed because *R* only tends to zero asymptotically in Eq 3B.

Population partitioning. Because cell death is negligible over the temporal scales of aggregation, we assumed that the aggregator–loner partitioning occurs instantaneously upon resource exhaustion. We explored 2 different scenarios:

- Coaggregation. Each patch is occupied by a homogenous mix of the 2 strains (S9 Fig). Upon resource exhaustion, the density of loners left behind by each of the 2 strains is determined from the pair-specific codevelopmental curve obtained via simulations of codevelopment using the spatially explicit developmental model above (S8A and S8C Fig). Whenever the composition of the mix at the end of a growth period does not coincide with any of the proportions sampled with codevelopmental simulations, we estimate the density of loners of each strain with a linear interpolation between the 2 closest points to the desired proportion. Finally, because both strains are homogeneously distributed across the whole patch of area 1, the number of loners of each strain *i*, $X_{i}^{L}$ (which is the variable of interest for our model), is identical to loner density. We obtain the number of aggregated cells of each strain as the difference between that strain’s population size upon resource exhaustion (immediately before the population partitioning) and its number of loners.
- Segregated development. Each patch is occupied by the 2 strains, but they do not mix; we therefore assume that they occupy a fraction of the patch area equal to that strain’s proportion (S9 Fig). Upon resource exhaustion, the density of loners left behind by each of the 2 strains is determined from simulations of the developmental model above under clonal conditions. To obtain the number of loners, we multiply the density by the fraction of the patch (of area 1) occupied by that strain. We then obtain the number of aggregated cells as in the coaggregating scenario.

After the population partitioning occurs, based on experimental measures that consistently find an 80:20 spore/stalk ratio within *D*. *discoideum* fruiting bodies formed under identical conditions [58], we multiply the number of aggregated cells of each strain *i* by the same constant factor *s* = 0.8 that reflects the effect of spore–stalk cell differentiation. This operation yields the number of reproductive spores, $X_{i}^{SP}$.

Starvation. The population partitioning is followed by a starvation period of length *T*_*st*_, in which both aggregated and nonaggregated cells die but at different rates. Spores die at a constant and low rate *δ*, whereas loners have a survival probability, *S*, that decays with time and reaches zero at a maximum survival time *T*_*sur*_. Analogous with [12], we fit this maximum survival time, as well as the functional shape of the survivorship curve using experimental data [15], to obtain

$$S(t)=\frac{e^{-{\left(\mu t\right)}^{\varsigma }}-e^{-{\left(\mu T_{sur}\right)}^{\varsigma }}}{1-e^{-{\left(\mu T_{sur}\right)}^{\varsigma }}},$$ (4)

where *μ* is the rate of decrease of the survival probability and *ς* is a parameter that modulates the decay of *S* with time. At the end of the starvation phase, we obtain the populations of surviving loners and spores of each strain *i* as

$$X_{i}^{L}(t+T_{st})=X_{i}^{L}(t)S(T_{st}),$$ (5A)

$$X_{i}^{SP}(t+T_{st})=X_{i}^{SP}(t)e^{-\delta T_{st}}.$$ (5B)

The lengths of the starvation periods *T*_*st*_ are either constant (deterministic environments, in which the length of each starvation period coincides with its mean value) or drawn from an exponential distribution with a mean ${\bar{T}}_{st}$ that gives the expected length of the starvation periods. We label deterministic environments using the length of their starvation periods *T*_*st*_ and stochastic environments using their expected value ${\bar{T}}_{st}.\;{\bar{T}}_{st}$ (or *T*_*st*_ in deterministic environments) is a measure for the environmental quality. Lower values of ${\bar{T}}_{st}$ represent better environments in which pulses of resources arrive more frequently on average; larger values of ${\bar{T}}_{st}$ represent worse environments in which resources recover less frequently. Each starvation phase ends with the arrival of the next resource pulse of size *R*_0_. We assume that all loners have the same strain-independent viability (i.e., that different strains do not have differential loner mortality); therefore, upon resource replenishment, all surviving loner cells start reproducing immediately, following Eq 3. Spores take an additional time *T*_*ger*_ to germinate [59], during which they continue to die at rate *δ*. At the end of the germination time, not all spores become reproducing cells; spores have a probability *ω* of germinating successfully [10]. Therefore, we multiply the total number of spores by a constant factor *ω* to obtain the fraction of the population of spores that become viable cells and start reproducing according to Eqs. 3A and 3B.

We repeat this sequence of growth–starvation cycles until one of the strains becomes extinct. We then record the winning strain for each patch and the time to extinction for the loser (proxy for transient alpha diversity). Once we have obtained the winner in each patch, we calculate the variability in the winner across different patches, *Φ* (proxy for stationary beta diversity), as

$$\Phi =\frac{\#-|{\#}_{w}-{\#}_{b}|}{\#},$$ (6)

where # = 10^4^ is the total number of patches and #_*w*_ and #_*b*_ are the number of patches dominated by the worse and the better aggregator, respectively, in the stationary state. From Eq 6, it follows that *Φ* varies between 0 and 1 (*Φ* = 1 when #_*w*_ = #_*b*_ and *Φ* = 0 when #_*w*_ = # or #_*b*_ = #). Since the extinction times and the noise in the extinction times and *Φ* vary depending on the pair of strains and the environmental conditions, mean values are taken over a varying number of independent realizations of the model to optimize computational efficiency. A summary of the model parameterization is provided in S1 Table.

*Measure of bias in spore production*. We obtain the bias in spore production as the difference between the frequency of the worse aggregator in the population of spores and its frequency in the initial population of plated cells,

$$B=\frac{{SP}_{w}}{{SP}_{w}+{SP}_{b}}-\frac{\rho_{0,w}}{\rho_{0,w}+\rho_{0,b}},$$

where *SP* is the number of spores and *ρ*_0_ the total density of plated cells. The subscripts *w* and *b* refer to the worse and the better aggregator, respectively. For each of the pairs, we calculated the number of spores from the density of loners, *SP*_*w*(*b*)_ = *s A*(Π_*w*(*b*)_*ρ*_0_ – *ρ*_*L*,*w*(*b*)_). Notice that *B* does not depend on the choice of *s* (fraction of aggregated cells that become spores) and *A* (area of the simulated aggregation territory), but it does depend on the initial cell density because of the plateau in loner density.

Data were deposited in the Dryad repository: https://doi.org/10.5061/dryad.zs7h44j4k [60].

## Supporting information

S1 Fig Loner cells are denser at the border of aggregation territories than in the immediate vicinity of aggregation centers. (A) Photo of an aggregation territory of *D*. *discoideum* strain NC85.2 aggregating on 3% agar (see S2 Video). Background is subtracted and image is thresholded to show areas with cell cover in black. Black area in the center is the aggregate about to enter the slug stage. All other black speckles are loner cells. (B) Proportion of the area of each of the concentric annuli in (A) that is covered by cells is plotted as a histogram. Dashed bar denotes the central circle where the aggregate has formed. Loner cell coverage is denser farther from aggregation center. (C) The same histogram as in (B) was created for every aggregation territory in S2 Video. For each aggregation territory, the loner cell coverage of the annulus at distance 0.2 mm (green) from the aggregation center and the loner cell coverage of the annulus at distance 0.5 mm (blue) were plotted. The annulus farther from the aggregation center always has higher cell coverage (*p* < 0.0002).
(TIFF)

S2 Fig Experimental loner spatial distributions. (A) Representative loner position maps are shown for each of the 3 strains (NC28.1 in blue, NC85.2 in red, and NC34.1 in gray) plated on 3% agar. The position of each cell is plotted such that darker regions represent regions densely packed with loners. (B) Characteristic loner spatial patterns for each strain are expressed as the probability distribution of local cell densities (see Materials and Methods). Broader peaks and fatter distribution tails (such as for NC34.1) correspond to more heterogeneously distributed loner cells.
(TIFF)

S3 Fig Experimental loner counts. (A) Loners in regions with varying loner densities were algorithmically counted and plotted against manual (by eye) counts for those same regions. Dashed line = automatic and manual counts coincide. The dispersion around the line is a measure of the counting error. (B) Cell counts in experiments realized with dilutions from a same cell suspension. Cell densities were below the aggregation threshold. Dashed line = linear regression with intercept anchored at zero. The inclination is a measure of the cell density of the initial suspension, and the dispersion around the regression line is a measure of the error introduced whenever a dilution is made. (C–K) Loner counts are shown as a function of initial cell plating densities for each of the 3 strains and each of the 3 substrate agar concentrations. For initial plating densities above 7.5 × 10^4^ cells/cm^2^, aggregation occurs for all strains and substrates. To test whether above this critical cell density, the decision to aggregate is context-independent, those samples with high initial plating densities (solid circles) were used to fit linear Gaussian models with zero intercept (dashed lines). These zero-intercept models were contrasted to linear Gaussian models with a free-intercept parameter (solid lines). ΔAIC, the difference in AIC between the zero-intercept and free-intercept models, shows that the latter outperformed the former for all substrates and strains, indicating that the decision to aggregate is context-dependent. Moreover, the inclines of the best-fitting linear models are not significantly different from zero for all but the best aggregating conditions (strain NC28.1 on 2% agar substrates) and even then only weakly positive. This indicates that loner densities plateau at high initial plating densities. AIC, Akaike Information Criterion
(TIFF)

S4 Fig Schematic of the developmental model. We formulated an individual-based model approach in which cells can be in 3 possible internal states: preaggregating, *P*; aggregating, *A*; and multicellular, *M*. Each state has different properties, listed in the blue boxes. The transitions between states occur only in one direction, as indicated by the gray arrows. The *P*-to-*A* transition is based on quorum sensing and it occurs at a strain-specific rate, *λ*; for each time step *dt*, if the density of signals is above the strain-specific sensitivity threshold, *P-*cells have a probability *λdt* of becoming *A*-cells. The transition from aggregation to multicellularity is entirely based on movement, and it occurs when cells arrive at the aggregation center
(TIF)

S5 Fig Spatially explicit numerical results for clonal aggregation. (A, B) Loner density versus initial cell density when (A) strains differ in *λ*/*ν* with fixed *κ* = 500 or (B) strains differ in *κ* with fixed *λ* = 1 and *ν* = 12 μm/min. *D* = 10^−7^. (C) Probability density function for the presence of loners; the aggregation center is at the center of the system. The histogram is computed using the spatial positions of loners from 100 independent realizations of the model with *D* = 3 × 10^−8^, *ρ*_0_ = 3 × 10^5^, *λ* = 1, *κ* = 400. (D, E) Loner density versus diffusion coefficient when (D) strains differ in *λ*/*ν* with fixed *κ* = 500 and (E) strains differ in *κ* with fixed *λ* = 1 and *ν* = 12 μm/min. (F, G) Schematic representation of the reduction in the regions in which signal density is above the strain-specific sensitivity threshold as a result of reducing the diffusion coefficient. Dashed red lines delineate the regions in which signal density is above a strain-specific sensitivity threshold. Color code for the cells and the concentration of signals as in Fig 2A–2D. In (A–E), nonspecified parameters and units are as in S1 Table.
(TIF)

S6 Fig Model results for codevelopment. For a systematic exploration of the outcome of pairwise developmental interactions within the three-dimensional strain-specific parameter space (*γ*, *θ*, *λ*), strains in each mix are labeled according to their relative value of the sensitivity threshold, *θ*. We use the subindex *lt*, standing for “low threshold,” to label strain-specific parameter values of the strain with the lowest *θ*, and the subindex *ht*, standing for less sensitive, to label strain-specific parameter values of the strain with the highest *θ*. (A) *γ*_*ht*_/*γ*_*lt*_ − *θ*_*ht*_/*θ*_*lt*_ parameter space (*θ*_*ht*_/*θ*_*lt*_ > 1 by definition). The thick-dashed lines trace 2 transects of the parameter space in which *κ*_*ht*_ = *κ*_*lt*_ (lower line) and *κ*_*ht*_ = 4*κ*_*lt*_ (upper line). Densities of mixed loners are shown in (B–D) for the parameter values along the lower line and in (E–G) for parameter values along the upper line. Specific parameter relationships are indicated by the positions of the squares, whose color is maintained in the mixed-loner curves (B–G). (B–D) *κ*_*ht*_ = *κ*_*lt*_ = 600, with *θ*_*ht*_ = 300 and *θ*_*lt*_ = 300 (darker brown), *θ*_*lt*_ = 150 (brown), and *θ*_*lt*_ = 100 (lighter brown); (B) *λ*_*ht*_ = *λ*_*lt*_ = 1; (C) *λ*_*ht*_ = 2, *λ*_*lt*_ = 1; (D) *λ*_*ht*_ = 1, *λ*_*lt*_ = 2. (E–G) *κ*_*ht*_ = 800 with *θ*_*ht*_ = 400 and *κ*_*lt*_ = 200 with *θ*_*lt*_ = 25, 33, 50, 100, 200, and 400 from top to bottom curve (red to black); (E) *λ*_*ht*_ = *λ*_*lt*_ = 1; (F) *λ*_*ht*_ = 2, *λ*_*lt*_ = 1; (G) *λ*_*ht*_ = 1, *λ*_*lt*_ = 2. Dashed lines in (B–G) indicate the null hypothesis. Model parameterization shown in S1 Table with *D =* 10^−7^ and *ρ*_0_ = 3 × 10^5^. Averages taken over 100 independent model realizations.
(TIF)

S7 Fig Statistical analysis of nonlinearity in mixed-strain experiments. (A–C) Maximum likelihood analysis. (A) Gray points = experimental mixed-loner densities (see Fig 4B). Black curve = expected loner densities for the maximum likelihood estimate of shape parameter *a* (see Materials and Methods). Blue areas = envelopes for the loner density curves for the confidence intervals defined by likelihood ratios of 2, 8, and 16, from darker to lighter. (B) Negative log–likelihood profile for the shape parameter *a* of the model with the best AIC. Blue areas = confidence intervals defined as in (A). (C) ΔAIC, the difference in AIC between a given model and the best model in the candidate set. Blue values = the 2 best-fitting models. (D, E) Bootstrapping analysis. For each of the 5 strain mix proportions, empirical distributions were bootstrapped, and 50,000 data sets were constructed. (D) Gray lines = piecewise linear regressions of 20 of these resampled data sets. Black line = the mean of all resampled data sets. Error bars = standard errors. (E) For each resampled data set, a linear regression was performed using only the pure strain experiments, and another linear regression was performed using only the mixed-strain experiments. The difference between these inclinations is a measure of the nonlinearity of the data set. Black line shows the probability density function of these inclination differences. Red line at zero marks linearity (*p* = 0.033). AIC, Akaike Information Criterion
(TIFF)

S8 Fig Model results for the effects of codevelopment on individual strains. As a consequence of developmental interactions, 2 given strains diverge (A, B) and 2 given strains converge (C, D) in their partitioning behavior. (A, C) Simulations of the individual-based model, *D* = 10^−7^. (B, D) Analytical approximations to (A, C) obtained in the limit *D* → ∞ (Eqs. 2.31 and 2.35 in S1 Text) qualitatively recapitulate the behavior of mixed loners and of the loners of each strain. Parameterization: *γ*_*w*_ = 0.5, *θ*_*w*_ = 400 (*κ*_*w*_ = 800), λ_*w*_ = λ_*b*_ = 1, *κ*_*b*_ = 200 with (A, B) *γ*_*b*_ = 0.125 and (C, D) *γ*_*b*_ = 1. *w* = worse aggregator; *b* = better aggregator. Remaining parameters are as in S1 Table. The color code for each strain corresponds to Fig 5.
(TIF)

S9 FigSchematic of the competition model.The model consists of a sequence of growth–starvation cycles. During growth, cells consume a shared pulse of resources and divide; during starvation, loners and aggregated cells die at different rates. The length of the starvation periods *T*_*st*_ can be either fixed (deterministic environments, defined by *T*_*st*_) or drawn from an exponential distribution (stochastic environments, defined by the mean starvation time ${\bar{T}}_{st}$). Upon resource exhaustion (at the end of the growth period), the population partitions into aggregators and loners according to our population-partitioning model. We compare 2 scenarios: coaggregation, in which co-occurring strains codevelop and loner densities are obtained from codevelopment curves (for example, as in S8 Fig), or segregation, in which strains are assumed to not mix and loners are derived from each strain’s clonal development partitioning.(TIF)

S10 Fig Bias in spore production due to different partitioning behaviors and coaggregation. The bias in spore number is calculated as the difference between the frequency of spores and the initial frequency of the strain (see Materials and Methods). (A, C) Bias resulting from the developmental model, at the end of a single aggregation event. (B, D) Bias resulting from the ecological model after each growth–starvation (aggregation) cycle in deterministic environments with *T*_*sur*_ < *T*_*st*_ (chosen to ensure no loner survival). The intensity of the color of the symbols (for both red and blue curves) indicates the index of each aggregation event in the time series according to the color bars. Darker symbols correspond to measures performed earlier in the pairwise competition.
(TIF)

S1 Table Parameterization of the 2 theoretical models. CMF, conditioned medium factor.
(PDF)

S1 Text Analytical calculations for a simplified version of the model. (PDF)

S1 Video Strain NC85.2 is shown aggregating in a 2% agar substrate. (M4V)

S2 Video Strain NC85.2 is shown aggregating in a 3% agar substrate. (M4V)

S3 Video Strain NC28.1 is shown aggregating in a 2% agar substrate. (M4V)

S4 Video Strain NC28.1 is shown aggregating in a 3% agar substrate. (M4V)

S5 Video Closeup of S2 Video in a region that ended up with high loner density. (AVI)

S6 Video Closeup of S2 Video in a region that ended up with low loner density. (AVI)
