# Supplementary figures and images for: Eco-evolutionary significance of “loners”
Source: PLoS Biol. 2020 Mar 19;18(3):e3000642. doi: 10.1371/journal.pbio.3000642 (PMC7081983; doi:10.1371/journal.pbio.3000642)

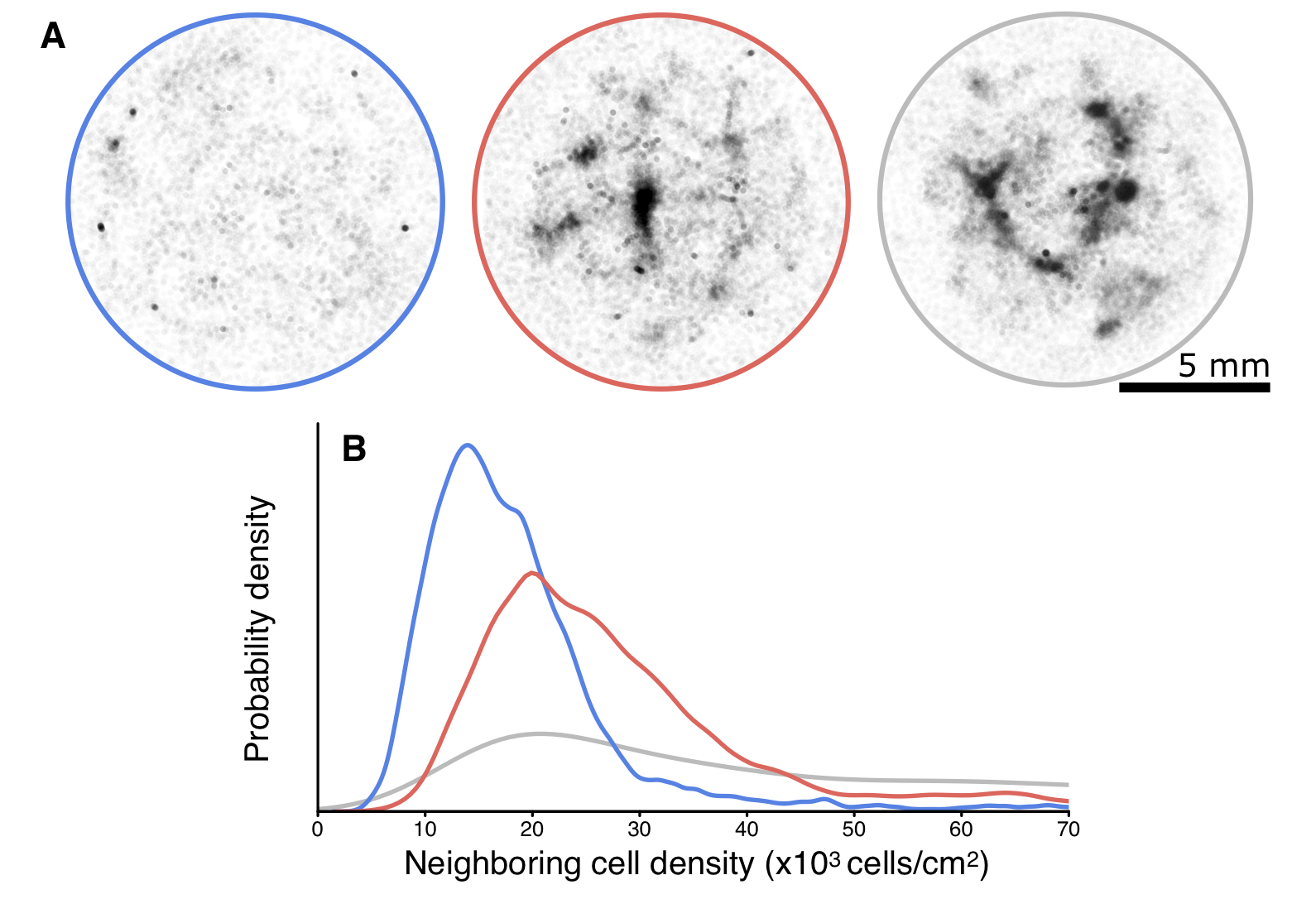

Supplement: S2 Fig — (A) Representative loner position maps are shown for each of the 3 strains (NC28.1 in blue, NC85.2 in red, and NC34.1 in gray) plated on 3% agar. The position of each cell is plotted such that darker regions represent regions densely packed with loners. (B) Characteristic loner spatial patterns for each strain are expressed as the probability distribution of local cell densities (see Materials and Methods). Broader peaks and fatter distribution tails (such as for NC34.1) correspond to more heterogeneously distributed loner cells. (TIFF) [file pbio.3000642.s002.tiff]

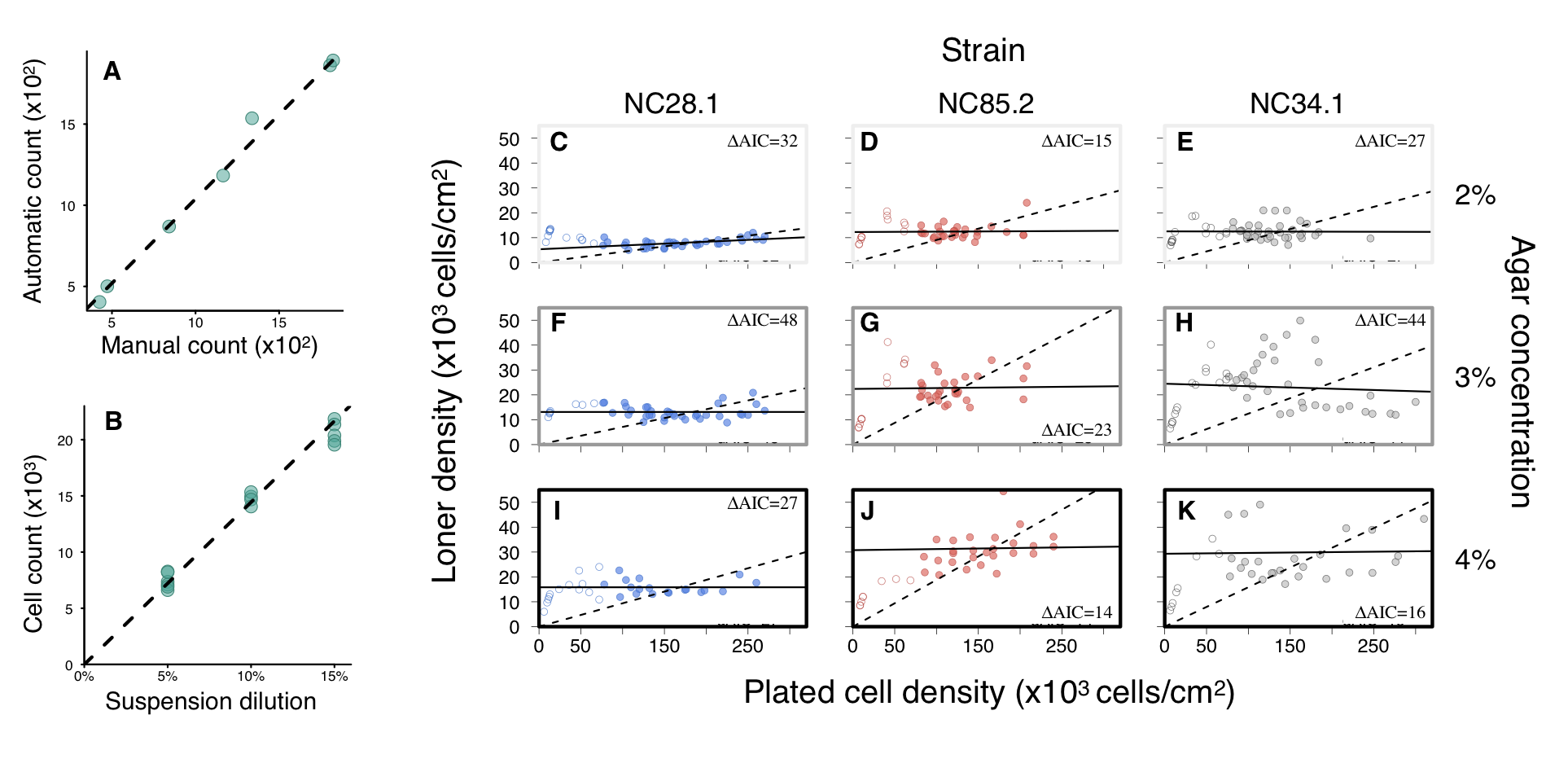

Supplement: S3 Fig — (A) Loners in regions with varying loner densities were algorithmically counted and plotted against manual (by eye) counts for those same regions. Dashed line = automatic and manual counts coincide. The dispersion around the line is a measure of the counting error. (B) Cell counts in experiments realized with dilutions from a same cell suspension. Cell densities were below the aggregation threshold. Dashed line = linear regression with intercept anchored at zero. The inclination is a measure of the cell density of the initial suspension, and the dispersion around the regression line is a measure of the error introduced whenever a dilution is made. (C–K) Loner counts are shown as a function of initial cell plating densities for each of the 3 strains and each of the 3 substrate agar concentrations. For initial plating densities above 7.5 × 10^4^ cells/cm2, aggregation occurs for all strains and substrates. To test whether above this critical cell density, the decision to aggregate is context-independent, those samples with high initial plating densities (solid circles) were used to fit linear Gaussian models with zero intercept (dashed lines). These zero-intercept models were contrasted to linear Gaussian models with a free-intercept parameter (solid lines). ΔAIC, the difference in AIC between the zero-intercept and free-intercept models, shows that the latter outperformed the former for all substrates and strains, indicating that the decision to aggregate is context-dependent. Moreover, the inclines of the best-fitting linear models are not significantly different from zero for all but the best aggregating conditions (strain NC28.1 on 2% agar substrates) and even then only weakly positive. This indicates that loner densities plateau at high initial plating densities. AIC, Akaike Information Criterion (TIFF) [file pbio.3000642.s003.tiff]

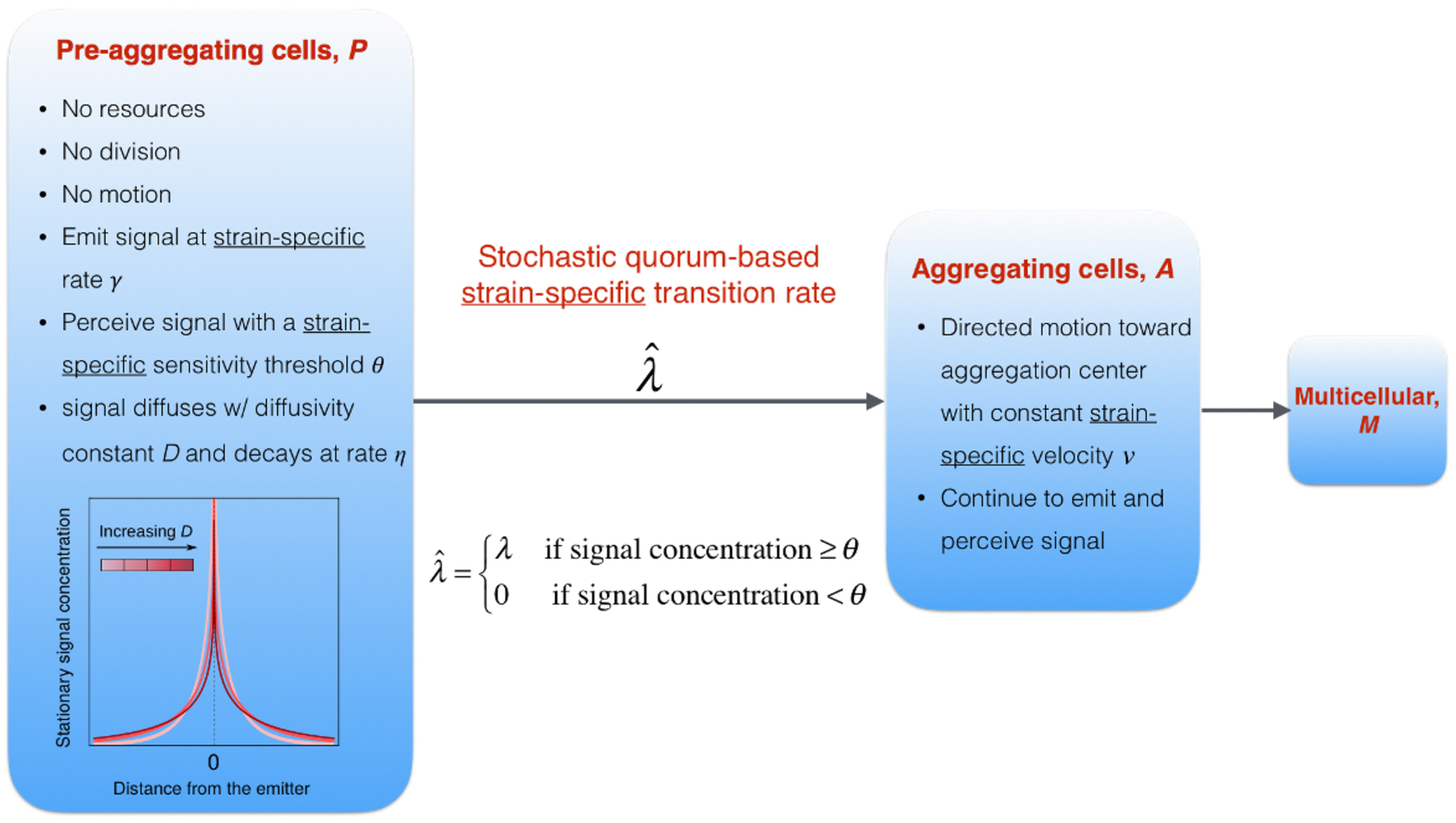

Supplement: S4 Fig — We formulated an individual-based model approach in which cells can be in 3 possible internal states: preaggregating, P; aggregating, A; and multicellular, M. Each state has different properties, listed in the blue boxes. The transitions between states occur only in one direction, as indicated by the gray arrows. The P-to-A transition is based on quorum sensing and it occurs at a strain-specific rate, λ; for each time step dt, if the density of signals is above the strain-specific sensitivity threshold, P-cells have a probability λdt of becoming A-cells. The transition from aggregation to multicellularity is entirely based on movement, and it occurs when cells arrive at the aggregation center (TIF) [file pbio.3000642.s004.tif]

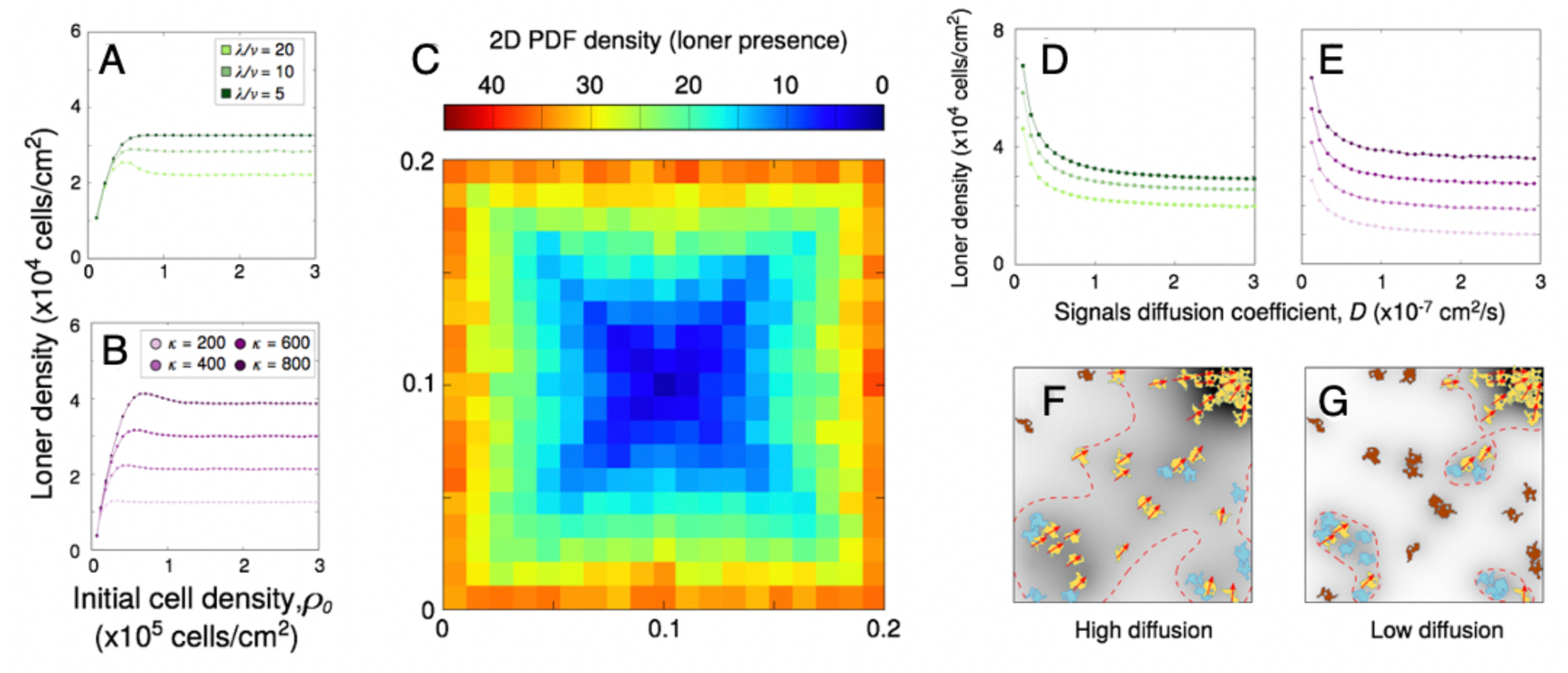

Supplement: S5 Fig — (A, B) Loner density versus initial cell density when (A) strains differ in λ/ν with fixed κ = 500 or (B) strains differ in κ with fixed λ = 1 and ν = 12 μm/min. D = 10−7. (C) Probability density function for the presence of loners; the aggregation center is at the center of the system. The histogram is computed using the spatial positions of loners from 100 independent realizations of the model with D = 3 × 10^−8^, ρ0 = 3 × 10^5^, λ = 1, κ = 400. (D, E) Loner density versus diffusion coefficient when (D) strains differ in λ/ν with fixed κ = 500 and (E) strains differ in κ with fixed λ = 1 and ν = 12 μm/min. (F, G) Schematic representation of the reduction in the regions in which signal density is above the strain-specific sensitivity threshold as a result of reducing the diffusion coefficient. Dashed red lines delineate the regions in which signal density is above a strain-specific sensitivity threshold. Color code for the cells and the concentration of signals as in Fig 2A–2D. In (A–E), nonspecified parameters and units are as in S1 Table. (TIF) [file pbio.3000642.s005.tif]

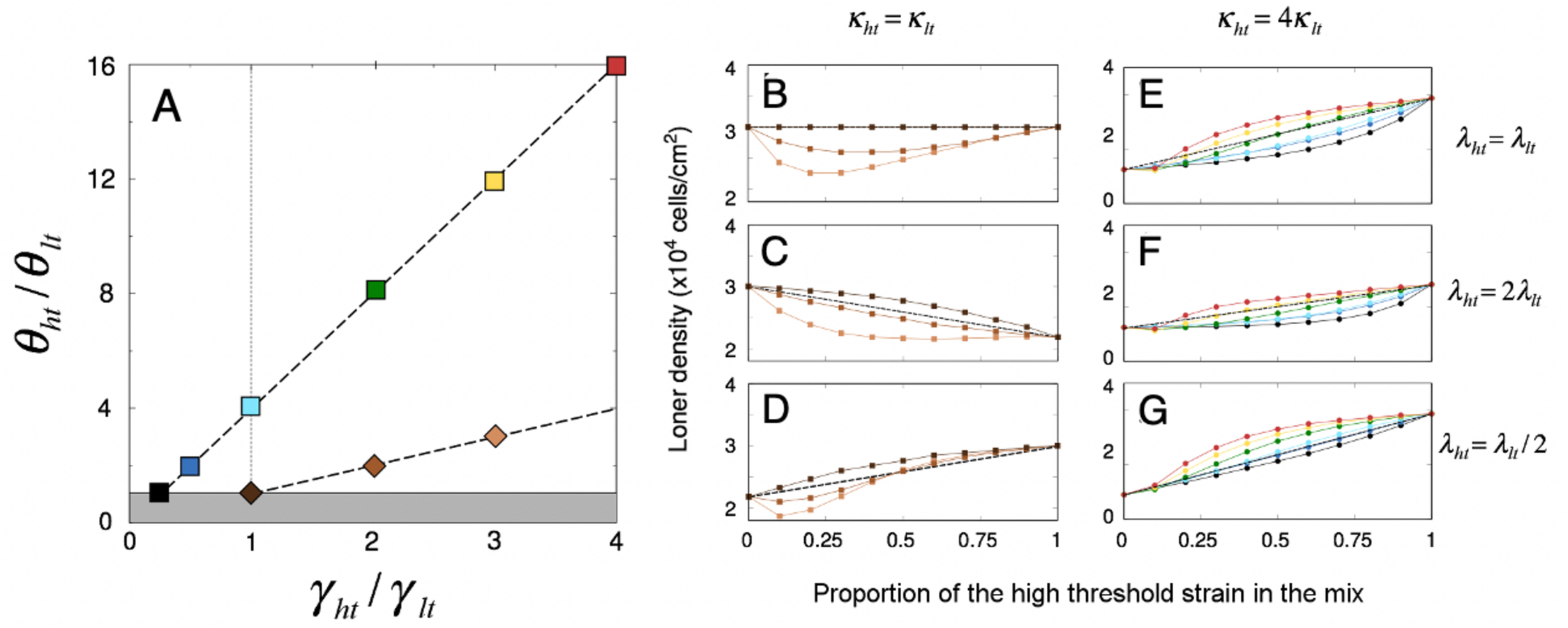

Supplement: S6 Fig — For a systematic exploration of the outcome of pairwise developmental interactions within the three-dimensional strain-specific parameter space (γ, θ, λ), strains in each mix are labeled according to their relative value of the sensitivity threshold, θ. We use the subindex lt, standing for “low threshold,” to label strain-specific parameter values of the strain with the lowest θ, and the subindex ht, standing for less sensitive, to label strain-specific parameter values of the strain with the highest θ. (A) γht/γlt − θht/θlt parameter space (θht/θlt > 1 by definition). The thick-dashed lines trace 2 transects of the parameter space in which κht = κlt (lower line) and κht = 4κlt (upper line). Densities of mixed loners are shown in (B–D) for the parameter values along the lower line and in (E–G) for parameter values along the upper line. Specific parameter relationships are indicated by the positions of the squares, whose color is maintained in the mixed-loner curves (B–G). (B–D) κht = κlt = 600, with θht = 300 and θlt = 300 (darker brown), θlt = 150 (brown), and θlt = 100 (lighter brown); (B) λht = λlt = 1; (C) λht = 2, λlt = 1; (D) λht = 1, λlt = 2. (E–G) κht = 800 with θht = 400 and κlt = 200 with θlt = 25, 33, 50, 100, 200, and 400 from top to bottom curve (red to black); (E) λht = λlt = 1; (F) λht = 2, λlt = 1; (G) λht = 1, λlt = 2. Dashed lines in (B–G) indicate the null hypothesis. Model parameterization shown in S1 Table with D = 10−7 and ρ0 = 3 × 10^5^. Averages taken over 100 independent model realizations. (TIF) [file pbio.3000642.s006.tif]

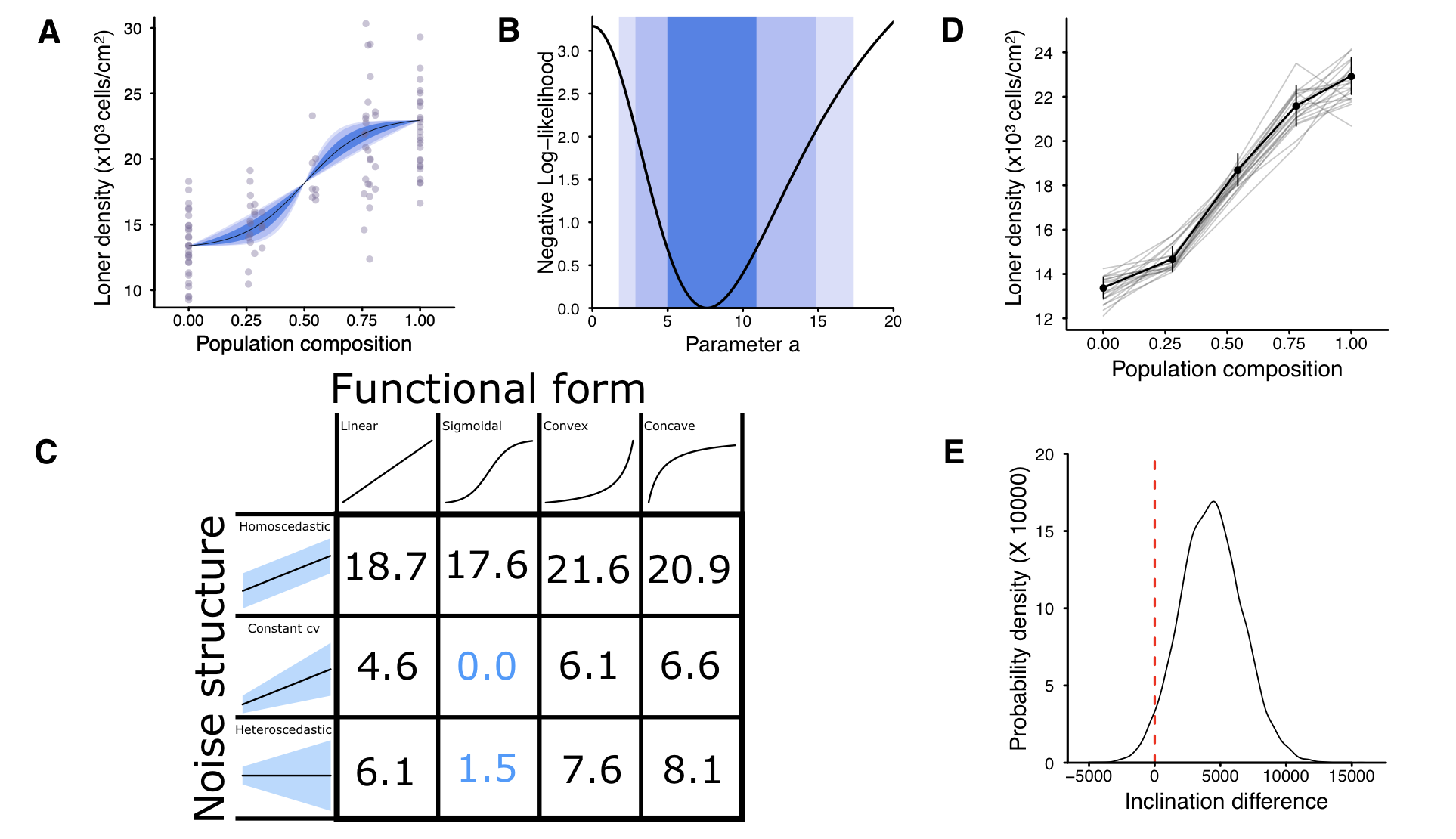

Supplement: S7 Fig — (A–C) Maximum likelihood analysis. (A) Gray points = experimental mixed-loner densities (see Fig 4B). Black curve = expected loner densities for the maximum likelihood estimate of shape parameter a (see Materials and Methods). Blue areas = envelopes for the loner density curves for the confidence intervals defined by likelihood ratios of 2, 8, and 16, from darker to lighter. (B) Negative log–likelihood profile for the shape parameter a of the model with the best AIC. Blue areas = confidence intervals defined as in (A). (C) ΔAIC, the difference in AIC between a given model and the best model in the candidate set. Blue values = the 2 best-fitting models. (D, E) Bootstrapping analysis. For each of the 5 strain mix proportions, empirical distributions were bootstrapped, and 50,000 data sets were constructed. (D) Gray lines = piecewise linear regressions of 20 of these resampled data sets. Black line = the mean of all resampled data sets. Error bars = standard errors. (E) For each resampled data set, a linear regression was performed using only the pure strain experiments, and another linear regression was performed using only the mixed-strain experiments. The difference between these inclinations is a measure of the nonlinearity of the data set. Black line shows the probability density function of these inclination differences. Red line at zero marks linearity (p = 0.033). AIC, Akaike Information Criterion (TIFF) [file pbio.3000642.s007.tiff]

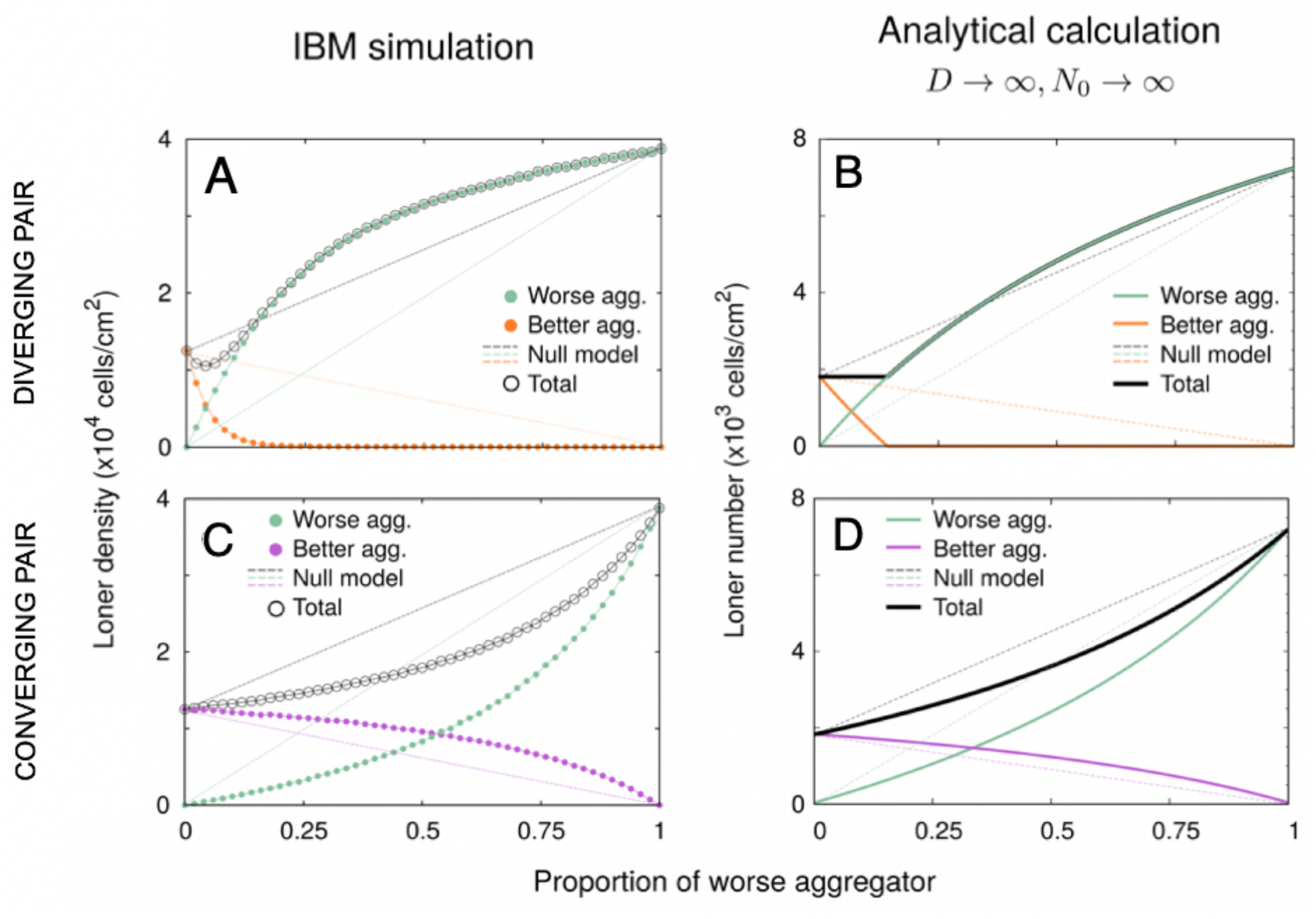

Supplement: S8 Fig — As a consequence of developmental interactions, 2 given strains diverge (A, B) and 2 given strains converge (C, D) in their partitioning behavior. (A, C) Simulations of the individual-based model, D = 10−7. (B, D) Analytical approximations to (A, C) obtained in the limit D → ∞ (Eqs. 2.31 and 2.35 in S1 Text) qualitatively recapitulate the behavior of mixed loners and of the loners of each strain. Parameterization: γw = 0.5, θw = 400 (κw = 800), λw = λb = 1, κb = 200 with (A, B) γb = 0.125 and (C, D) γb = 1. w = worse aggregator; b = better aggregator. Remaining parameters are as in S1 Table. The color code for each strain corresponds to Fig 5. (TIF) [file pbio.3000642.s008.tif]

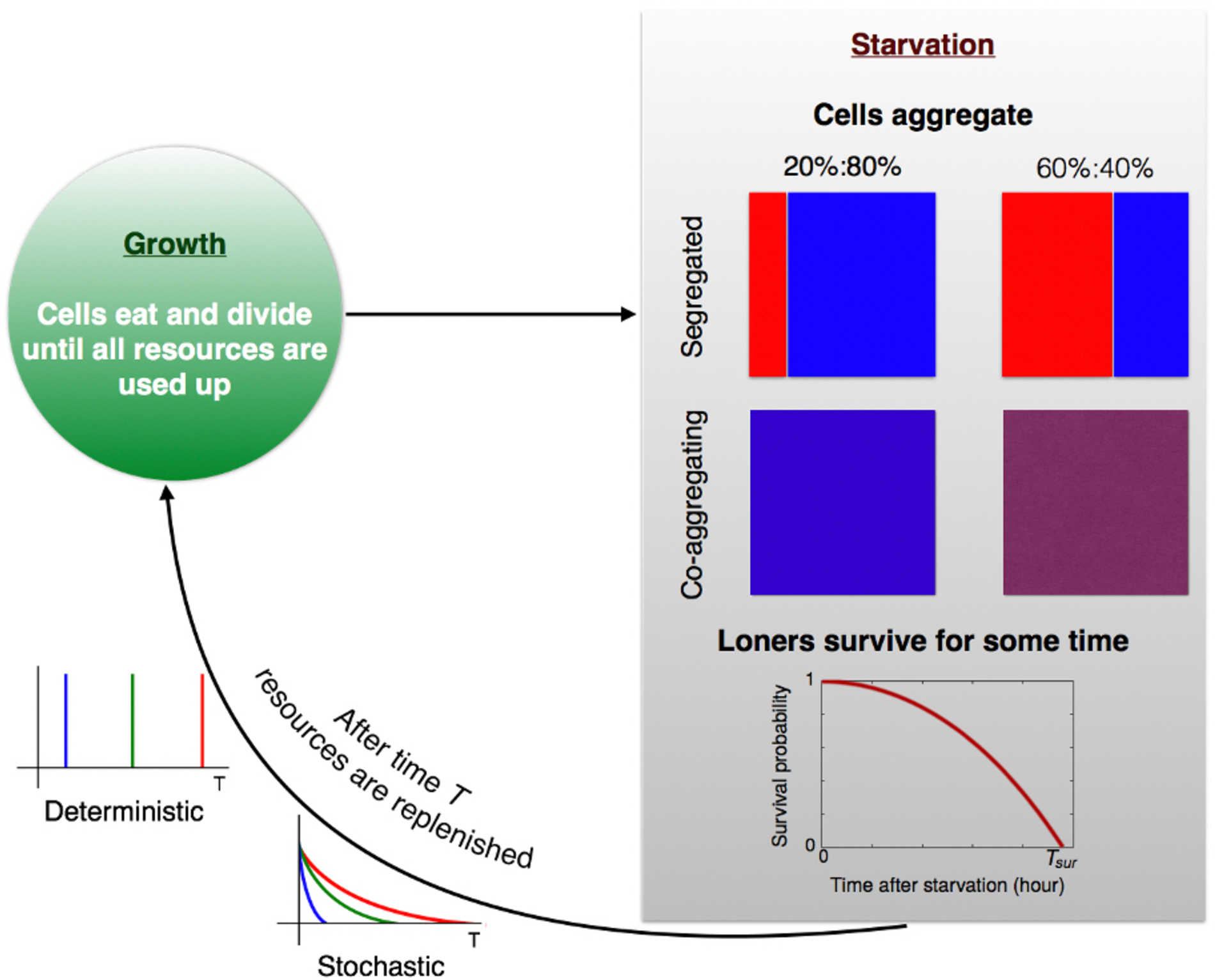

Supplement: S9 Fig — The model consists of a sequence of growth–starvation cycles. During growth, cells consume a shared pulse of resources and divide; during starvation, loners and aggregated cells die at different rates. The length of the starvation periods Tst can be either fixed (deterministic environments, defined by Tst) or drawn from an exponential distribution (stochastic environments, defined by the mean starvation time T¯st). Upon resource exhaustion (at the end of the growth period), the population partitions into aggregators and loners according to our population-partitioning model. We compare 2 scenarios: coaggregation, in which co-occurring strains codevelop and loner densities are obtained from codevelopment curves (for example, as in S8 Fig), or segregation, in which strains are assumed to not mix and loners are derived from each strain’s clonal development partitioning. (TIF) [file pbio.3000642.s009.tif]

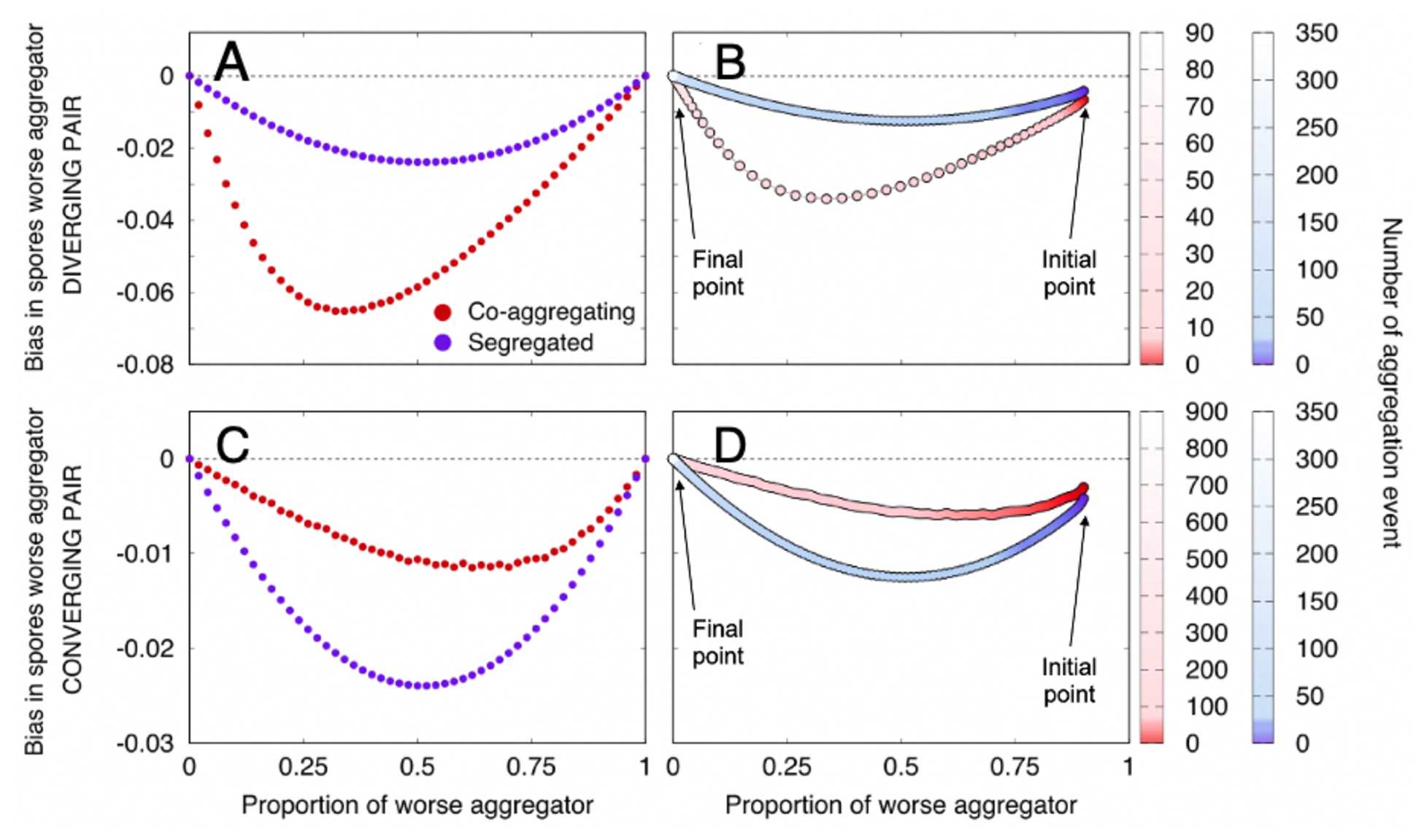

Supplement: S10 Fig — The bias in spore number is calculated as the difference between the frequency of spores and the initial frequency of the strain (see Materials and Methods). (A, C) Bias resulting from the developmental model, at the end of a single aggregation event. (B, D) Bias resulting from the ecological model after each growth–starvation (aggregation) cycle in deterministic environments with Tsur < Tst (chosen to ensure no loner survival). The intensity of the color of the symbols (for both red and blue curves) indicates the index of each aggregation event in the time series according to the color bars. Darker symbols correspond to measures performed earlier in the pairwise competition. (TIF) [file pbio.3000642.s010.tif]
