# Supplementary material for: Eco-evolutionary significance of “loners”
Source: PLoS Biol. 2020 Mar 19;18(3):e3000642. doi: 10.1371/journal.pbio.3000642 (PMC7081983; doi:10.1371/journal.pbio.3000642)
Supplement: S1 Table — CMF, conditioned medium factor. (PDF) [file pbio.3000642.s011.pdf]

Theory					Experiment		
	Symbol	Definition	Units	Model value	Experimental observable	Exp. value	Ref.
**Population partitioning model**	λ	Strain specific *P*-to-*A* transition rate	hour^{-1}	Varied	--		
	θ	Strain-specific sensitivity threshold	Kg/cm^{2}	Varied	--		
	γ	Strain-specific signaling rate	Kg/hour	Varied	--		
	v	Strain-specific *A*-cells velocity	μm/min	Varied around 12	Velocity of aggregating cells	12μm/min	[53]
	$\tilde{v}$	Rescaled cell velocity ($D \rightarrow \infty$ limit; see Sup. Information)	hour^{-1}	Varied	--		
	N_0	Initial number of *P*-cells	# cells	Varied	--		
	η	Decay constant of the signal	min^{-1}	1.2	Parameter used for model fitting.		
	D	Signal diffusion coefficient	cm^{2}/s	Varied	cAMP diff. coeff. (2% agar)<br>CMF diff. coeff. (water film)	4.4x10^{-6} cm^{2}/s<br>8x10^{-7} cm^{2}/s	[54–56]
**Resource competition model**	μ	Decrease rate of survival probability	hour^{-1}	2x10^{-3}	Number of alive/moving loners versus time	2x10^{-3} hour^{-1}	Fitting from [14]
	ς	Resistance to starvation parameter	--	2		2	
	T_{sur}	Loner maximum lifespan	hour	240		240 hour	
	T_{ger}	Spore germination time	hour	4	Mean germination time 1-3 days old spores	4-8 hour	[58]
	δ	Spore death rate	hour^{-1}	2x10^{-4}	--		
	c	Maximum division rate	hour^{-1}	0.173	Doubling time	4 hour	[50]
	s	Spore:stalk ratio	--	0.8	Spore:stalk proportion	~ 80:20	[57]
	ω	Spore germination success	--	0.63	Germination efficiency	0.63	[9]
	R_0, X_0	Food pulse size; normalized initial population size	# cells	3x10^{5}	--		
	$R_{1/2}$	Resources consumption half saturation constant	--	0.1R_0	--		
	$\bar{T}_{st}$	Mean starvation time	hour	Varied	--		
