# Supplementary material for: Eco-evolutionary significance of “loners”
Source: PLoS Biol. 2020 Mar 19;18(3):e3000642. doi: 10.1371/journal.pbio.3000642 (PMC7081983; doi:10.1371/journal.pbio.3000642)
Supplement: S1 Text — (PDF) [file pbio.3000642.s012.pdf]

Supplementary Text for

Eco-evolutionary significance of 'loners'

F.W Rossine[§], R. Martinez-Garcia[§], A.E. Sgro, T. Gregor* & C.E. Tarnita*

Contents

1 Calculation of stationary signal profile **1**

2 Analytical treatment of the developmental model in the spatially-implicit limit $D \to \infty$ **3**

2.1 Analytical results for the non-spatial limit $\tilde{v} \to \infty$ 4

2.2 Analytical results when $\tilde{v} = k\lambda$ or $\tilde{v} = \lambda/k$ 5

2.3 Analytical results in the large population limit $N_0 \to \infty$ 5

2.4 Analytical results for co-development of two strains with same λ and $\tilde{v} = 2\lambda$ 7

2.4.1 $N_0 \to \infty$ limit: the shape of co-development curves 8

1 Calculation of stationary signal profile

We assume that cells are punctual sources that release signal at a constant strain-specific rate γ. The signal has a spontaneous decay rate η and a diffusion coefficient D. Given these conditions, the equation that governs the spatiotemporal evolution of signal density, $\sigma(x, y; t)$, is

$$\frac{\partial \sigma(x, y; t)}{\partial t} = D\nabla\sigma(x, y; t) - \eta\sigma(x, y; t), \tag{1.1}$$

The first term on the right side of Eq. (1.1) accounts for the diffusion of signal and the second term for its spontaneous decay. Here, we first solve the stationary limit ($\partial_t = 0$) of Eq. (1.1) in an infinite domain, imposing as boundary conditions the facts that cells continuously release signals and that signal density goes to zero when the distance from the emitting cell tends to infinity. Subsequently, we discuss the effect of considering a finite integration domain with periodic boundary conditions.

Due to the radial symmetry of the problem, we transform Eq. (1.1) to polar coordinates, in which the partial differential equation in (x, y) becomes an ordinary differential equation in the radial coordinate r that indicates the distance to the source of the signal,

$$D\left(\frac{d^2\sigma(r)}{dr^2} + \frac{1}{r}\frac{d\sigma(r)}{dr}\right) - \eta\sigma(r) = 0. \tag{1.2}$$

Since the position of the emitter, $r = 0$, is a singular point of Eq. (1.2), we will first assume that cells have a finite radius $\tilde{r}$ and then take the limit $\tilde{r} \to 0$. After the transformation to polar coordinates, and assuming

a finite radius for the cell, the boundary conditions can be written as,

$$\sigma_{\tilde{r}}(r\to\infty) = 0, \tag{1.3}$$

$$-2\pi\tilde{r}D\left.\frac{d\sigma_{\tilde{r}}(r)}{dr}\right|_{r=\tilde{r}} = \gamma. \tag{1.4}$$

Eq. (1.3) imposes the finiteness of the density, and Eq. (1.4) imposes that the amount of mass released through the boundary of the cell per unit time has to be constant and equal to the strain-specific emission rate γ. The subscript $\tilde{r}$ in the densities accounts for the finite radius of the cells.

Equation (1.2) is the modified Bessel equation of order zero, and its general solution can be expressed as

$$\sigma_{\tilde{r}}(r) = AI_0\left(\sqrt{\frac{\eta}{D}}r\right) + BK_0\left(\sqrt{\frac{\eta}{D}}r\right), \tag{1.5}$$

where I_0 and K_0 are the zero order modified Bessel functions of the first, respectively second, kind. From the boundary condition of Eq. (1.3), it follows that $A = 0$, since I_0 diverges when its argument tends to infinity. B is calculated from the second boundary condition, Eq. (1.4),

$$B = \frac{\gamma}{2\pi\tilde{r}\sqrt{\eta D}K_1\left(\sqrt{\frac{\eta}{D}}\tilde{r}\right)}, \tag{1.6}$$

where K_1 is the first order modified Bessel function of the second kind and we have used that $K_0'(r) = -K_1(r)$.

Inserting Eq. (1.6) into (1.5), the stationary signal profile produced by a source of finite radius $\tilde{r}$ is,

$$\sigma_{\tilde{r}}(r) = \frac{\gamma}{2\pi\tilde{r}\sqrt{\eta D}K_1\left(\sqrt{\frac{\eta}{D}}\tilde{r}\right)}K_0\left(\sqrt{\frac{\eta}{D}}r\right). \tag{1.7}$$

Finally, to obtain the profile generated by a punctual source, we take the limit $\tilde{r}\to 0$ in Eq. (1.7),

$$\sigma(r) = \frac{\gamma}{2\pi D}K_0\left(\sqrt{\frac{\eta}{D}}r\right). \tag{1.8}$$

The solution provided by Eq. (1.8) assumes an infinite system size, whereas we perform numerical simulations of the developmental model on a finite domain of lateral length ℓ with periodic boundary conditions. To impose periodic boundary conditions is equivalent to considering that the simulated finite domain corresponds to a tile embedded into an infinite lattice in which each tile is a mirroring image of the focal domain. The signal density within the focal tile is obtained by adding over the contributions of all other tiles. However, since our numerical simulations only explore a range of diffusion coefficients in which $\sigma(\ell/2)\approx 0$, we can truncate the sum over tiles at the nearest neighbors of the focal one. This is equivalent to calculating distances to the position of each emitting cell, $(x_{\mathrm{em}}, y_{\mathrm{em}})$, in each spatial coordinate:

$$r_x = \begin{cases} |x-x_{\mathrm{em}}| & \text{if } |x-x_{\mathrm{em}}|\le \ell/2 \\ \ell-|x-x_{\mathrm{em}}| & \text{if } |x-x_{\mathrm{em}}| > \ell/2 \end{cases}$$

$$r_y = \begin{cases} |y-y_{\mathrm{em}}| & \text{if } |y-y_{\mathrm{em}}|\le \ell/2 \\ \ell-|y-y_{\mathrm{em}}| & \text{if } |y-y_{\mathrm{em}}| > \ell/2 \end{cases}$$

The total distance is then given by the radial coordinate r, as $r = \sqrt{r_x^2 + r_y^2}$.

2 Analytical treatment of the developmental model in the spatially-implicit limit $D \to \infty$

The spatially-implicit limit of the individual based population-partitioning model consists of disregarding the spatial effects introduced by a finite signal diffusion coefficient (i.e. the limit $D \to \infty$), but still accounting for cell movement at a finite velocity. To this end, we map cell movement into a stochastic transition in cell state from aggregating to being multicellular; the rate of this transition is related to cell velocity, v.

First, we calculate the stationary signal density profile produced by each cell in the limit $D \to \infty$. Unlike in the low D case explored in the spatially-explicit simulations, in which periodic boundary conditions were implemented considering only the nearest neighbors of the focal tile, now, since the signal spreads infinitely far, we need to include the contribution of an infinite number of tiles. This results in each cell generating a homogeneous signal distribution within the focal tile, $\sigma^{\mathrm{H}} = \mathcal{M}/\ell^2$. $\mathcal{M}$ is the mass of signal that is being released by each cell in the stationary limit, which can be obtained by integrating Eq. (1.8) over the entire range of distances,

$$\mathcal{M} = \int_0^\infty r\sigma(r)dr = \frac{\gamma}{2\pi\eta}. \tag{2.1}$$

Due to the conservation of the total population size N_0 (since demographic events are neglected on the temporal scales of aggregation), the state of the system is fully determined by the sizes of two of the three subpopulations (P, A, and M cells). We choose the number of cells in the P-state, N_P, and in the A-state, N_A, as state variables. The number of cells in the M state, $N_M(t)$ (i.e., the size of the multicellular aggregate) can then be obtained from $N_P(t) + N_A(t) + N_M(t) = N_0$.

In order for the aggregation process to be initiated at all, a quorum must be met by the initial population (all of which are P-cells), i.e. we must have $N_0\sigma^{\mathrm{H}} > \theta$. In the absence of a quorum, all initial cells remain as loners and therefore the total loner number is $L = N_0$. If there is an initial quorum, then P-cells turn into A-cells at rate λ; A-cells continue to emit signal while they move in the direction of the aggregate. As A-cells eventually join the aggregate, they stop signaling and therefore the amount of signal in the system continues to decrease. P-cells continue to become A-cells at rate λ only if the total signal density $[N_P(t) + N_A(t)]\,\sigma^{\mathrm{H}}$ remains above the strain-specific sensitivity threshold, θ. The P-to-A transition rate as a function of time is thus given by

$$\hat{\lambda}(t) = \lambda\Theta\left(\sigma^{\mathrm{H}}\left[N_P(t) + N_A(t)\right] - \theta\right), \tag{2.2}$$

where Θ is the Heaviside function, which takes value 1 for non-negative arguments and 0 for negative arguments. Therefore, omitting the temporal dependence in $\hat{\lambda}$, N_A and N_P,

$$\hat{\lambda} = \begin{cases} \lambda & \text{if } N_P + N_A \geq \theta/\sigma^{\mathrm{H}} \\ 0 & \text{otherwise.} \end{cases} \tag{2.3}$$

The rate at which A-cells stick to the aggregate and become M-cells can be approximated by the inverse of the time needed to cover the mean distance to the aggregate at a velocity v, i.e.

$$\tilde{v} = \frac{v}{< d >} \tag{2.4}$$

where $< d >$ is a characteristic spatial scale of the aggregation territory (mean distance to the aggregation center). For simplicity, we will fix $< d >= 1$ in the following and refer to $\tilde{v}$ as a rescaled velocity.

Therefore, the aggregation process can be mapped to a sequence of two stochastic reactions, each of which occurs at a different rate,

$$P \xrightarrow{\hat{\lambda}(t)} A \qquad\qquad A \xrightarrow{\tilde{v}} M, \tag{2.5}$$

This stochastic process is fully described by a master equation, which gives the temporal evolution of the probability $g(N_P, N_A; t)$ of finding the system in a state (N_P, N_A) at time t,

$$\frac{\partial g(N_P,N_A;t)}{\partial t} = \tilde{v}(N_A+1)g(N_P, N_A+1;t) + \hat{\lambda}(N_P+1)g(N_P+1, N_A-1;t) \tag{2.6}$$
$$- \left(N_P\hat{\lambda} + N_A\tilde{v}\right) g(N_P, N_A; t) \tag{2.7}$$

To simplify the notation, the temporal dependence in N_A, N_P and $\hat{\lambda}$ has been omitted.

Following standard procedures, from the Master equation (2.7) we can derive a system of coupled ordinary differential equations for the mean value of each subpopulation size,

$$\begin{aligned} \dot{p}(t) &= -\hat{\lambda}p(t) \\ \dot{a}(t) &= \hat{\lambda}p(t) - \tilde{v}a(t) \end{aligned} \tag{2.8}$$

where $p(t)$ and $a(t)$ are the mean values of N_P, respectively N_A, at time t. The dot over a and p on the left side of the equation indicates a time derivative. System (2.8) can be solved analytically, using that initially all cells are in the pre-aggregation state, i.e. $p(0) = N_0$, $a(0) = 0$. Then

$$p(t) = N_0 \mathrm{e}^{-\hat{\lambda}t} \tag{2.9}$$
$$a(t) = \frac{\hat{\lambda}N_0}{\tilde{v}-\hat{\lambda}}\left(\mathrm{e}^{-\hat{\lambda}t} - \mathrm{e}^{-\tilde{v}t}\right). \tag{2.10}$$

Since the ultimate objective of this approximation is to obtain analytical expressions for the loner-aggregator partitioning behavior, an important observable is the time τ at which the decaying signal density exactly equals the strain-specific sensitivity threshold. τ can thus be obtained by solving

$$\theta = \sigma^{\mathrm{H}}[p(\tau) + a(\tau)] = \frac{\sigma^{\mathrm{H}}N_0}{\tilde{v}-\lambda}\left(\tilde{v}\mathrm{e}^{-\lambda\tau} - \lambda\mathrm{e}^{-\tilde{v}\tau}\right), \tag{2.11}$$

where we have used the fact that $\hat{\lambda}(\tau) = \lambda$ according to Eq. (2.2). Since aggregating cells also contribute to the pool of signal, τ does not represent the aggregation time; after a time τ, any A-cell in the system will continue to move towards the aggregate at rate $\tilde{v}$ until $a(\tau + \Delta t) = 0$. However, importantly, τ gives the time at which the last $P - A$ transition occurs. Therefore, all cells that are still in the P-state at time τ will remain as loners and we can find the total number of loners as

$$L = \begin{cases} p(\tau) & \text{if} \quad N_0\sigma^{\mathrm{H}} > \theta \qquad \text{(i.e. a quorum is met)} \\ N_0 & \text{otherwise} \end{cases} \tag{2.12}$$

Henceforth we will focus on the former case, when aggregation does get initiated.

In general, we can not solve for τ in Eq. (2.11) and therefore we can not determine the number of loners analytically. Below, we try to circumvent this problem by looking at a few special cases.

2.1 Analytical results for the non-spatial limit $\tilde{v} \to \infty$

In this limit, cells spend an infinitesimally short time in the A state and therefore $p(t) + a(t) \to N_0\mathrm{e}^{-\lambda/t}$. To obtain τ we

then solve $\sigma^{\mathrm{H}}N_0\mathrm{e}^{-\lambda/\tau} = \theta$, which gives $\tau = \ln\left(\frac{\sigma^{\mathrm{H}}N_0}{\theta}\right)/\lambda$. Then, from Eq. (2.12), the number of loners, when there is a quorum for aggregation, is

$$L = \exp(-\lambda/\tau) = \theta/\sigma^{\mathrm{H}}. \tag{2.13}$$

Therefore, in this limit, λ gives the time scale of the aggregation but it has no effect on the number of loners, which is equal to the sensing-to-signal ratio.

2.2 Analytical results when $\tilde{v} = k\lambda$ or $\tilde{v} = \lambda/k$

In the special case $\tilde{v} = 2\lambda$, using the change of variables $y = \exp(-\lambda\tau)$, (2.11) becomes a quadratic equation from which y and thus τ can be obtained,

$$y^2 - 2y + \frac{\theta}{N_0\sigma^{\text{H}}} = 0. \tag{2.14}$$

Given the definition of y, Eq. (2.14) only has physical meaning in the domain $y \in (0, 1]$. Within that interval, if a quorum exists (i.e. $N_0 > \theta/\sigma^{\text{H}}$), Eq. (2.14) has a single root, which determines the number of loners $p(\tau)$ according to Eq. (2.9) and the definition of y:

$$L = N_0 y = N_0 \left(1 - \sqrt{1 - \frac{\theta}{N_0\sigma^{\text{H}}}}\right). \tag{2.15}$$

In the limit $N_0 \to \infty$, Eq. (2.15) tends to $\theta/(2\sigma^{\text{H}})$, as predicted by Eq. (1) in the main text (also Eq. (2.22) below).

In the other special case, $\tilde{v} = \lambda/2$, Eq. (2.11) becomes again Eq. (2.14) using the change of variables $y = \exp(-\tilde{v}t)$. Thus, if there is a quorum for aggregation (i.e. $N_0 > \theta/\sigma^{\text{H}}$) the number of loners is

$$L = N_0 y^2 = N_0 \left(1 - \sqrt{1 - \frac{\theta}{N_0\sigma^{\text{H}}}}\right)^2, \tag{2.16}$$

which tends to 0 in the limit $N_0 \to \infty$, as predicted by the phase separation defined in Eq. (2.22 below (see also Box 1 in the main text).

In general, the changes of variables introduced here, $y = \exp(-\lambda t)$ and $y = \exp(-\tilde{v}t)$, will turn Eq. (2.11) into a polynomial equation of degree n provided that $\tilde{v} = n\lambda$ or $\tilde{v} = \lambda/n$. If the root of such a polynomial within the interval $y \in (0, 1]$ can be obtained, then an expression for the number of loners as a function of the initial population N_0 is accessible. In Fig. 1a, we show the two cases obtained in Eqs. (2.15) and (2.16) together with the $\tilde{v} = 4\lambda$ case. For the latter, the equivalent to Eq. (2.14) is a 4-th degree polynomial, whose root in the interval $y \in (0, 1]$ we obtained using *Mathematica 11.1*. For completion, we also show the non-spatial limit $\tilde{v} \to \infty$ in Eq. (2.13)

2.3 Analytical results in the large population limit $N_0 \to \infty$

In the limit of infinitely large initial population size, a quorum is always met. Therefore, from Eq. (2.12), the number of loners is

$$L = \lim_{N_0 \to \infty} p(\tau) = \lim_{N_0 \to \infty} N_0 \mathrm{e}^{-\lambda\tau}. \tag{2.17}$$

At the end of this section, we prove that p is a positive and monotonically decreasing function of N_0; therefore L always exists and is greater than or equal to zero. This also implies that $\lim\limits_{N_0 \to \infty} \mathrm{e}^{-\lambda\tau} = 0$ (otherwise L would not be finite); applying L'Hôpital's rule to Eq. (2.17), we obtain

$$L = \frac{N_0^2 \tilde{v}\sigma^{\text{H}} \left[\mathrm{e}^{-2\lambda\tau} - \mathrm{e}^{-\lambda\tau}\mathrm{e}^{-\tilde{v}\tau}\right]}{\theta(\tilde{v} - \lambda)}, \tag{2.18}$$

where we have used Eq. (2.25) for the derivative of τ with respect to N_0. Finally, defining $Q \equiv \lim\limits_{N_0 \to \infty} N_0 \mathrm{e}^{-\tilde{v}\tau}$

and rearranging terms, we get

$$L = Q + \frac{\theta(\tilde{v} - \lambda)}{\sigma^{\rm H}\tilde{v}}. \tag{2.19}$$

To obtain an independent expression for L we need another, non-redundant relationship between L and Q. This can be obtained by first rearranging terms in Eq. (2.11),

$$N_0\left[\frac{\mathrm{e}^{-\lambda\tau}}{\lambda} - \frac{\mathrm{e}^{-\tilde{v}\tau}}{\tilde{v}}\right] = \frac{(\tilde{v} - \lambda)\theta}{\tilde{v}\lambda\sigma^{\rm H}}, \tag{2.20}$$

and then taking the limit $N_0 \to \infty$ in Eq. (2.20),

$$\frac{L}{\lambda} - \frac{Q}{\tilde{v}} = \frac{(\tilde{v} - \lambda)\theta}{\tilde{v}\lambda\sigma^{\rm H}}. \tag{2.21}$$

Solving for L in Eqs. (2.18) and (2.21), we find

$$L = \begin{cases} 2\pi\eta\ell^2\ \frac{\theta}{\gamma}\left(1 - \frac{\lambda}{\tilde{v}}\right) & \text{if}\ \ \lambda \leq \tilde{v} \\ 0 & \text{if}\ \ \lambda > \tilde{v}, \end{cases} \tag{2.22}$$

where we have used the fact that $\sigma^{\rm H} = \frac{\gamma}{2\pi\eta\ell^2}$ (see Section 2). In the limit $N_0 \to \infty$, there is thus a phase separation given by the relative magnitudes of the P-to-A and A-to-M transition rates.

Proof of the existence of L. In order to obtain in Eq. 2.22 the limit of $p(\tau)$ for infinite initial population sizes, we first need to prove that such a limit exists and is finite. To this end, we will first calculate the derivative of $p(\tau)$ with respect to N_0:

$$\frac{dp(\tau)}{dN_0} = \mathrm{e}^{-\lambda\tau} - N_0\lambda\tau'(N_0)\mathrm{e}^{-\lambda\tau}, \tag{2.23}$$

Although Eq. (2.11) for τ cannot be solved in general, we can obtain an analytical expression for the derivative of τ with respect to N_0 using implicit differentiation. We differentiate both sides of Eq. (2.11)

$$\left[\tilde{v}\mathrm{e}^{-\lambda\tau} - \lambda\mathrm{e}^{-\tilde{v}\tau}\right] + N_0\left[-\lambda\tilde{v}\tau'(N_0)\mathrm{e}^{-\lambda\tau} + \tilde{v}\lambda\tau'(N_0)\mathrm{e}^{-\tilde{v}\tau}\right] = 0, \tag{2.24}$$

and solving for $\tau'(N_0)$, we obtain

$$\tau'(N_0) = \frac{1}{\tilde{v}\lambda N_0}\left[\frac{\tilde{v}\mathrm{e}^{-\lambda\tau} - \lambda\mathrm{e}^{-\tilde{v}\tau}}{\mathrm{e}^{-\lambda\tau} - \mathrm{e}^{-\tilde{v}\tau}}\right], \tag{2.25}$$

which is always positive for any relationship between $\tilde{v}$ and λ. Using Eq. (2.25) in Eq. (2.23), we find

$$\frac{dp(\tau)}{dN_0} = \mathrm{e}^{-\lambda\tau}\left[1 - \frac{\mathrm{e}^{-\lambda\tau} - \frac{\lambda}{\tilde{v}}\mathrm{e}^{-\tilde{v}\tau}}{\mathrm{e}^{-\lambda\tau} - \mathrm{e}^{-\tilde{v}\tau}}\right] = \frac{\mathrm{e}^{-(\lambda+\tilde{v})\tau}\left(\frac{\lambda}{\tilde{v}} - 1\right)}{\mathrm{e}^{-\lambda\tau} - \mathrm{e}^{-\tilde{v}\tau}} \tag{2.26}$$

Since the numerator and the denominator of Eq. (2.26) have opposite signs for both $\lambda > \tilde{v}$ and $\tilde{v} > \lambda$, $dp(\tau)/dN_0$ is always negative. Thus, $p(\tau)$ is a decreasing function of N_0. Since p is a non-negative and decreasing function, the limit of $p(\tau)$ as N_0 tends to infinity exists and is always greater than or equal to zero. Importantly, due to the symmetry between p and $N_0\exp(-\tilde{v}\tau)$, the limit Q defined in the calculation of L also exists and has the same properties as L.

2.4 Analytical results for co-development of two strains with same λ and $\tilde{v} = 2\lambda$

In mixed development, we consider two strains defined by the set of strain-specific parameters $(\lambda, \theta, \gamma)$. λ and θ have been defined above, and γ determines the strain-specific signal density σ^{H} released by each cell. We use the term *high-threshold strain* and the notation ht for the strain with the higher signal sensitivity threshold and *low-threshold strain* (lt) for the one with the lower signal-sensitivity threshold. Thus, $\theta_{ht} > \theta_{lt}$.

If both strains have the same strain-specific λ, which is the case studied in this section, then the high-threshold strain also has the higher investment in loners (i.e. it is the worse aggregator) if

$$\frac{\theta_{ht}}{\sigma^{\mathrm{H}}_{ht}} > \frac{\theta_{lt}}{\sigma^{\mathrm{H}}_{lt}}. \tag{2.27}$$

To obtain the mixed loners, we generalize Eq. (2.11) to the two-strain case,

$$\sigma^{\mathrm{H}}_{ht}[p_{ht}(\tau_{ht}) + a_{ht}(\tau_{ht})] + \sigma^{\mathrm{H}}_{lt}[p_{lt}(\tau_{ht}) + a_{lt}(\tau_{ht})] = \theta_{ht}, \tag{2.28}$$

where τ_{ht} is the time at which the density of signal reaches the strain-specific sensitivity of the high-threshold strain. Therefore, for $t > \tau_{ht}$, $\hat{\lambda}_{ht} = 0$ and only cells of the low-threshold strain continue to aggregate ($\hat{\lambda}_{lt} \neq 0$).

Let Π be the proportion of the high-threshold strain in the mix; ΠN_0 is thus the initial population of the high-threshold strain, and $(1 - \Pi)N_0$ the initial population of the low-threshold strain. Substituting the expressions for $a(t)$ and $p(t)$ obtained in Eqs. (2.9) and (2.10), Eq. (2.28) becomes

$$\frac{N_0 \Pi \sigma^{\mathrm{H}}_{ht}}{\tilde{v} - \lambda_{ht}} \left[\tilde{v} \mathrm{e}^{-\lambda_{ht}\tau_{ht}} - \lambda_{ht} \mathrm{e}^{-\tilde{v}\tau_{ht}}\right] + \frac{N_0(1 - \Pi)\sigma^{\mathrm{H}}_{lt}}{\tilde{v} - \lambda_{lt}} \left[\tilde{v} \mathrm{e}^{-\lambda_{lt}\tau_{ht}} - \lambda_{lt} \mathrm{e}^{-\tilde{v}\tau_{ht}}\right] = \theta_{ht}. \tag{2.29}$$

τ_{ht} cannot be obtained from Eq. (2.29) in general. However, since we are assuming in this section that $\lambda_{ht} = \lambda_{lt} \equiv \lambda$ and $\tilde{v} = 2\lambda$, the change of variables $y = \exp(-\lambda\tau_{ht})$ turns Eq. (2.29) into a quadratic equation of the form

$$y^2 - 2y + \frac{\theta_{ht}}{N_0\Pi(\sigma^{\mathrm{H}}_{ht} - \sigma^{\mathrm{H}}_{lt}) + N_0\sigma^{\mathrm{H}}_{lt}} = 0, \tag{2.30}$$

that has only one root, y^*, in the interval $(0, 1]$. Using that root, we obtain the number of loners of the high-threshold strain,

$$L_{ht} = \Pi N_0 y^* = \Pi N_0 \left(1 - \sqrt{1 - \frac{\theta_{ht}}{N_0\Pi(\sigma^{\mathrm{H}}_{ht} - \sigma^{\mathrm{H}}_{lt}) + N_0\sigma^{\mathrm{H}}_{lt}}}\right) \tag{2.31}$$

After τ_{ht}, only cells of the low-threshold strain continue to aggregate. The number of loners left by the low-threshold strain is determined by the relationship between L_{ht}, σ^{H}_{ht}, and θ_{lt}:

1. If $\sigma^{\mathrm{H}}_{ht} L_{ht} \geq \theta_{lt}$, the loners of the high-threshold strain provide quorum for a full aggregation of the low-threshold strain and therefore $L_{lt} = 0$.

2. If $\sigma^{\mathrm{H}}_{ht} L_{ht} < \theta_{lt}$, the low-threshold strain stops aggregating at a time $\tau_{lt} > \tau_{ht}$, such that

$$\sigma^{\mathrm{H}}_{ht} L_{ht} + \sigma^{\mathrm{H}}_{lt} \left[p_{lt}(\tau_{lt}) + a_{lt}(\tau_{lt})\right] = \theta_{lt}. \tag{2.32}$$

In order to obtain the number of loners of the low-threshold strain, $p_{lt}(\tau_{lt})$, from Eq. (2.32), we repeat the steps followed to obtain the number of loners of the high-threshold strain. Inserting the expressions for $a(t)$ and $p(t)$ obtained in Eqs. (2.9) and (2.10) in Eq. (2.32), we obtain

$$\sigma^{\mathrm{H}}_{ht} L_{ht} + N_0(1 - \Pi)\sigma^{\mathrm{H}}_{lt} \left[2\mathrm{e}^{-\lambda\tau_{lt}} - \mathrm{e}^{-2\lambda\tau_{lt}}\right] = \theta_{lt}, \tag{2.33}$$

where we have already considered that $\lambda_{lt} \equiv \lambda$ and $\tilde{v} = 2\lambda$. Next, with the change of variables $y = \exp(-\lambda\tau_{lt})$, Eq. (2.33) becomes a quadratic equation of the form

$$y^2 - 2y + \frac{\theta_{lt} - \sigma^{\mathrm{H}}_{ht} L_{ht}}{N_0(1-\Pi)\sigma^{\mathrm{H}}_{lt}} = 0. \tag{2.34}$$

Eq. (2.34) has a single root y^* in the interval $(0, 1]$, from which we obtain the number of loners for the low-threshold strain

$$p_{lt}(\tau_{lt}) \equiv L_{lt} = (1-\Pi)N_0 y^* = (1-\Pi)N_0 \left[1 - \sqrt{1 - \frac{\theta_{lt} - \sigma^{\mathrm{H}}_{ht} L_{ht}}{N_0(1-\Pi)\sigma^{\mathrm{H}}_{lt}}}\,\right]. \tag{2.35}$$

Notice that taking the clonal-population limits, $\Pi = 1$ in Eq. (2.31) or $\Pi = 0$ in Eq. (2.35), we recover Eq. (2.15), which was obtained for the number of loners for single-strain development when $\tilde{v} = 2\lambda$.

2.4.1 $N_0 \to \infty$ limit: the shape of co-development curves

Eq. (2.31) and (2.35) provide a good approximation to the number of loners in co-development, and can be used to investigate how the total number of loners, $L_{ht} + L_{lt}$, versus Π curve responds to the signaling properties of the strains in the mix (sigmoidal, concave up or concave down). In particular, since we are assuming that both strains have the same P-to-A transition rate λ, we will focus on differences in strain signaling rate and sensitivity to signal, σ^{H} (or γ) and θ respectively. However, the dependence with N_0 in Eq. (2.31) and (2.35) makes these calculations unfeasible and we need to tackle them in the $N_0 \to \infty$ limit.

Taking the limit $N_0 \to \infty$ in Eq. (2.31) and (2.35), which give the number of loners of the high-threshold and the low-threshold strain respectively, we obtain,

$$L_{ht} = \frac{1}{2}\frac{\Pi\theta_{ht}}{\Pi\sigma^{\mathrm{H}}_{ht} + (1-\Pi)\sigma^{\mathrm{H}}_{lt}} \tag{2.36}$$

and

$$L_{lt} = \frac{\Pi(\theta_{ht} - 2\theta_{lt})\sigma^{\mathrm{H}}_{ht} + 2(1-\Pi)\theta_{lt}\sigma^{\mathrm{H}}_{lt}}{4\sigma^{\mathrm{H}}_{lt}\left[\Pi(\sigma^{\mathrm{H}}_{ht} - \sigma^{\mathrm{H}}_{lt}) - \sigma^{\mathrm{H}}_{lt}\right]} \tag{2.37}$$

From Eq. (2.36), we can obtain the critical population composition, Π^*, at which $\sigma^{\mathrm{H}}_{ht} L_{ht} = \theta_{lt}$ and hence the loners of the high-threshold strain provide a quorum for a complete aggregation of the low-threshold strain,

$$\Pi^* = \frac{2\theta_{lt}\sigma^{\mathrm{H}}_{lt}}{\sigma^{\mathrm{H}}_{ht}(\theta_{ht} - 2\theta_{lt}) + 2\theta_{lt}\sigma^{\mathrm{H}}_{lt}}. \tag{2.38}$$

Using the definition of σ^{H} as well as defining the nondimensional ratios between strain-specific signaling rates, $\alpha \equiv \gamma_{ht}/\gamma_{lt}$, and strain-specific sensitivity thresholds, $\beta \equiv \theta_{ht}/\theta_{lt}$ ($\beta > 1$ by definition), we can rewrite Eq. (2.38) in terms of only two parameters,

$$\Pi^* = \frac{2}{2 + \alpha(\beta - 2)}. \tag{2.39}$$

According to Eq. (2.39), Π^* is defined in the whole $\beta - \alpha$ parameter space except in the curve $\beta = 2(1 - 1/\alpha)$ where its denominator becomes zero (white-dashed curve in Fig. 2a). However, we will limit our analysis to $\beta < 2$ and $0 < \alpha < \infty$. We exclude $\beta < 1$ due to its definition, $\beta \equiv \theta_{ht}/\theta_{lt}$, whereas for $1 < \beta < 2$ we obtain unrealistic values of Π^* because θ_{ht} is to similar to θ_{lt} and the $N_0 \to \infty$ approximation is not accurate (Fig. 2a). For the rest of the $\alpha - \beta$ plane, we can evaluate Eq. (2.39) to obtain Π^* (Fig. 2a). Our results indicate that, when $\theta_{ht} >> \theta_{lt}$ and $\gamma_{ht} >> \gamma_{lt}$, that is $\beta \to \infty$ and $\alpha \to \infty$ simultaneously, full aggregation of the low-threshold strain requires a lower presence of high-threshold cells and therefore

$\Pi^* \to 0$.

Another important property of each two-strains co-development is the relation between the total number of loners and the composition of the mix (Fig. 4a of the main text and S5 Fig). Within this analytical treatment of the developmental model, we can limit the regions of the $\alpha - \beta$ space in which developmental interactions may be expected to return a sigmoidal response in the total number of loners similar to the experimental result (Fig. 4b of the main text). To have a sigmoidal curve, we need the number of loners to cross the null hypothesis (i.e., the linear model) at a mix composition, $\tilde{\Pi}$, such that, $0 < \tilde{\Pi} < 1$. This may occur in two situations:

$$L_{ht}(\tilde{\Pi}) + L_{lt}(\tilde{\Pi}) = [L_{ht}(1) - L_{lt}(0)]\tilde{\Pi} + L_{lt}(0) \quad \text{if} \quad \tilde{\Pi} < \Pi^*, \tag{2.40}$$

$$L_{ht}(\tilde{\Pi}) = [L_{ht}(1) - L_{lt}(0)]\tilde{\Pi} + L_{lt}(0) \quad \text{if} \quad \tilde{\Pi} > \Pi^*, \tag{2.41}$$

where we have written explicitly the dependence of the number of loners on Π. Notice that the first condition, Eq. (2.40), implies that the crossing point occurs for loner populations composed by both strains whereas Eq. (2.41) refers to the cases in which only the high-threshold strain leaves loners behind. Again, using Eq. (2.36) and (2.37) for the number of loners of each strain, we can obtain $\tilde{\Pi}$ from Eq. (2.40) and (2.41). From Eq. (2.40) ($\tilde{\Pi} < \Pi^*$) we obtain,

$$\tilde{\Pi}_1 = \frac{2(\alpha - \beta) - \alpha\beta(\alpha - 2)}{2(1 - \alpha)(\alpha - \beta)}, \tag{2.42}$$

which lies in the interval $(0, 1)$ if $\beta > 2$ and $\beta < \frac{2\alpha}{2+\alpha(\alpha-2)}$ (small colored region in lower part of Fig. 2b). Alternatively, from Eq. (2.40) ($\tilde{\Pi} > \Pi^*$) we obtain,

$$\tilde{\Pi}_2 = \frac{\alpha}{(1 - \alpha)(\alpha - \beta)}, \tag{2.43}$$

that lies in between 0 and 1 if $\beta > \frac{\alpha^2}{\alpha-1}$ (large colored region in the upper part of Fig. 2b). For fixed β, Π^* tends to zero as α increases, whereas $\tilde{\Pi}$ first quickly decreases from 1 and then increases again. The minimum value of $\tilde{\Pi}$ depends on β, with higher values of β resulting in lower values of $\tilde{\Pi}$. The shift in $\tilde{\Pi}$ when β remains constant and α increases is shown in the loner curves of Fig. 3.

In this particular case, in which $\lambda_{ht} = \lambda_{lt} = 2\tilde{v}$ and $\theta_{ht} \geq 2\theta_{lt}$, we have limited the strain-signaling and sensing properties in which co-development results in sigmoidal curves for the total number of loners. To conclude our analysis, we can explore whether these sigmoidal responses consistently belong to one of the modes of co-development introduced in the main text [case (i) or case(ii)] or whether they may belong to either of them. In case (i), the better aggregator, that is, the strain within the mix that leaves behind fewer loners in clonal development, becomes a worse aggregator in the presence of the second strain. Given the parameter constrains considered for these analytical calculations, the better aggregator corresponds to the lower-threshold strain. As a result of this shift in aggregation performance, the model prediction for its number of loners in co-development remains always above the null hypothesis. Conversely, the worse aggregator becomes a better aggregator in the presence of the second strain and the model prediction for the number of loners always remains below the null hypothesis (S7a Figure). Therefore, the model prediction for the number of loners is a concave down function for the better aggregator and a concave up function for the worse aggregator. For case (ii), we observe reversed trends. The better aggregator improves in aggregation, so the model prediction for the number of loners always remains below the null hypothesis and is concave up function. The worse aggregator worsens in aggregation and the model prediction for the number of loners is a concave down function that always remains above the null hypothesis (S7b Figure).

Therefore, studying the concavity of the model predictions for the number of loners of each subpopulation

(Eq. (2.36) and (2.37)), we can relate the shape of the curve for the total number of loners to a mode of co-development. The concavity of the model predictions for each subpopulation of loners can be calculated from the second derivative of Eq. (2.36) and (2.37) with respect to Π,

$$\frac{\partial^2 L_{ht}}{\partial \Pi^2} = \frac{\theta_{ht}\sigma^{\mathrm{H}}_{lt}(\sigma^{\mathrm{H}}_{lt} - \sigma^{\mathrm{H}}_{ht})}{[\Pi(\sigma^{\mathrm{H}}_{ht} - \sigma^{\mathrm{H}}_{lt}) + \sigma^{\mathrm{H}}_{lt}]^3}, \tag{2.44}$$

$$\frac{\partial^2 L_{lt}}{\partial \Pi^2} = \frac{\theta_{ht}\sigma^{\mathrm{H}}_{ht}(\sigma^{\mathrm{H}}_{ht} - \sigma^{\mathrm{H}}_{lt})}{[\Pi(\sigma^{\mathrm{H}}_{ht} - \sigma^{\mathrm{H}}_{lt}) + \sigma^{\mathrm{H}}_{lt}]^3}. \tag{2.45}$$

Because the denominator of Eq. (2.44) and (2.45) is always positive, the concavity of each curve is fully determined by the relationship between the signaling rates of the mixed strains. If $\sigma^{\mathrm{H}}_{lt} > \sigma^{\mathrm{H}}_{ht}$ ($\alpha > 1$), L_{ht} is concave up and L_{lt}, concave down. This scenario belongs to case (i). In contrast, if $\sigma^{\mathrm{H}}_{lt} < \sigma^{\mathrm{H}}_{ht}$ ($\alpha < 1$), the concavity of each curve is switched. L_{ht} is now concave down, L_{lt} concave up and we recover case (ii). Since sigmoidal developmental interactions are always found for $\alpha > 1$ ($\alpha = 1$ is marked by the vertical white-dashed and black-dotted lines in Fig. 2a and b respectively), we can conclude that, at least for this particular set of assumptions on strain-specific values of λ, $\tilde{v}$, and θ, sigmoidal curves in the total number of loners always arise from developmental interactions in case (ii).

Figures

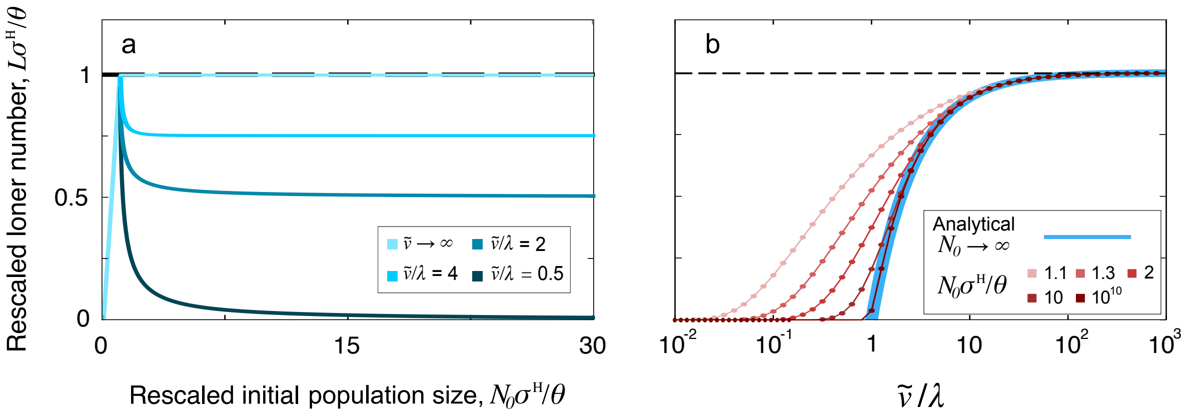

Figure 1: a) Rescaled number of loners as a function of the rescaled initial population size in the spatially-implicit limit ($D \to \infty$, v finite; Section 2.2) and the non-spatial limit ($D \to \infty$, $v \to \infty$; Section 2.1). b) Phase separation between perfectly (no loners) and imperfectly (loners) coordinated development in the spatially-implicit limit . Red curves are obtained via numerical integration of the system (2.8) for different initial population sizes; the blue-thick curve corresponds to the analytical result in the limit $N_0 \to \infty$ (Eq. (2.22), see also Box 1 in the main text).

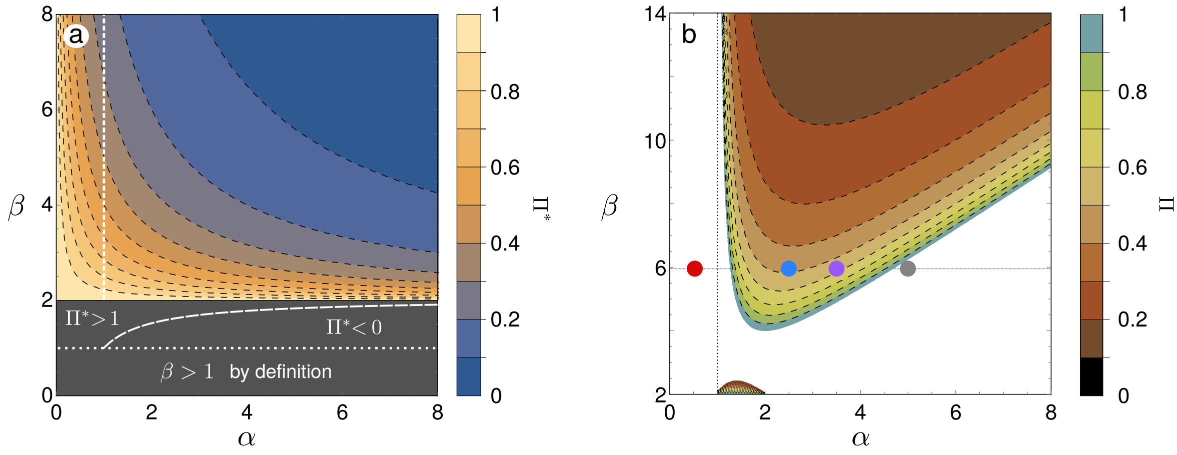

Figure 2: a) Contour plot for Π^* as a function of the signaling-rate and sensitivity-threshold ratios, α and β respectively. Gray regions are either inaccessible due to the definition of β or provide unrealistic results given the series of approximations done in the calculations. The vertical white-dashed line at $\alpha = 1$ indicates the transition from developmental interactions lying within case (i) when $\alpha < 1$ to interactions belonging to case (ii) when $\alpha > 1$. The white-dashed curve in the gray region indicates the existence of a singularity in Eq. (2.39). b) Contour plot for the population composition, $\tilde{\Pi}$, at which the model prediction and the null hypothesis for the total number of loners cross with each other. Each colored circle (from left to right: red, blue, purple, and gray) indicates the (α, β) coordinates used to obtain the curves shown in Fig. 3.

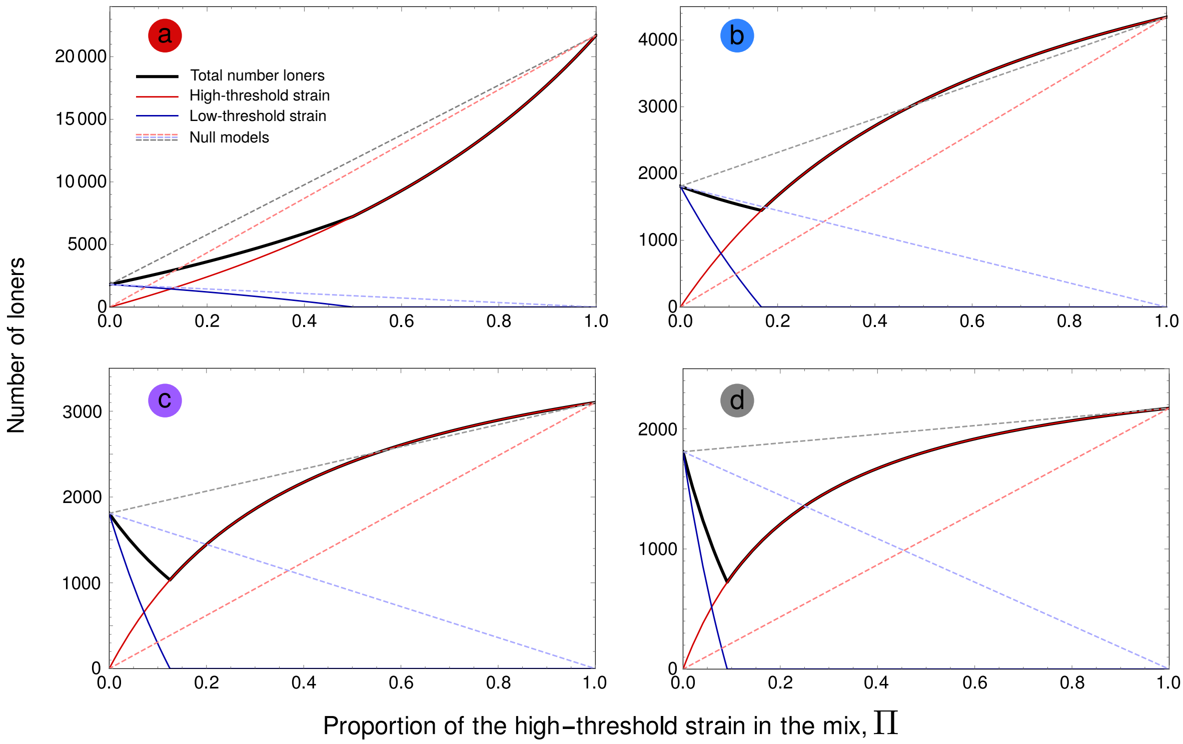

Figure 3: Analytical approximation to loner numbers obtained in the limits $D \to \infty$ and $N_0 \to \infty$ for $\beta = 6$ and $\alpha = 0.5$(a), 2.5(b), 3.5(c), 5(d). Other parameter assumptions, $\lambda_{ht} = \lambda_{lt} = 2\tilde{v}$.
